# 1-Octen-3-ol, a self-stimulating oxylipin messenger, can prime and induce defense of marine alga

**DOI:** 10.1186/s12870-019-1642-0

**Published:** 2019-01-22

**Authors:** Haimin Chen, Rui Yang, Juanjuan Chen, Qijun Luo, Xiaoshan Cui, Xiaojun Yan, William H. Gerwick

**Affiliations:** 1 Key Laboratory of Marine Biotechnology of Zhejiang Province, Ningbo University, Ningbo, Zhejiang, 315211 China; 2 Center for Marine Biotechnology and Biomedicine, Scripps Institution of Oceanography, University of California, La Jolla, San Diego, CA 92093 USA

**Keywords:** *Pyropia haitanensis*, 1-octen-3-ol, Volatile, Oxylipin, Self-catalyzing community messenger, Primed defense

## Abstract

**Background:**

Short chain oxylipins in plants as the main volatile organic carbon have been speculated to playing an important role for plant innate immunity, however, not yet intensively studied and far away established as the fully recognized algae defense signals.

**Results:**

The production of 1-octen-3-ol is self-amplified via the fatty acid-oxylipin metabolic cycle through positive feedback loop. Production of 1-octen-3-ol may act as a messenger that induces *P. haitanensis* to be in a “primed” state and ready for defense by upregulating the synthesis of methyl jasmonic acid, indole-3-acetic acid, and gibberellin A3. Production of these oxylipins also adjust the redox state in cells, resulting in host defense activation.

**Conclusions:**

We provide the first demonstration that 1-octen-3-ol from *P. haitanensis*, can act as a self-stimulating community messenger. The multiple effects of 1-octen-3-ol may explain why *P. haitanensis*, a very ancient lineage within plant kingdom, thrives in the niche of intertidal zones.

**Electronic supplementary material:**

The online version of this article (10.1186/s12870-019-1642-0) contains supplementary material, which is available to authorized users.

## Background

Compared with terrestrial plants, coastal marine plants are surrounded by mineral- and organic-rich seawater and thus constantly subjected to various potential biotic and abiotic stresses. Algae in the intertidal zone have adapted to the specific requirements of living attached to the benthos, and therefore, have developed complex defense strategies against pathogenic bacteria and herbivores, as well as environmental stresses [1].

Higher plants have sophisticated mechanisms by which to defend against threats from predators, pathogens or environmental stresses. A commonly employed strategy by many plant species is the production of short-chain oxylipins in response to specific external challenges. For example, in some cases this enables plants to specifically attract pollinators, leading to the activation of abiotic-stress related genes and protection against pathogenic bacteria and fungi [2, 3]. These volatile oxylipins can also act as airborne signals that mediate inter-plant communication, thus affecting both the challenged plant and its neighbors [4]. In addition, volatile oxylipins are involved in triggering “self-priming”, a state that prepares for a response to herbivore or pathogenic attacks without a large initial investment in defensive resources [5]. A large body of evidence has indicated that algae can also produce a surprisingly diverse array of volatile oxylipins. However, studies have focused primarily on the existence and structure of these oxylipins [6, 7], although there have been some investigations into their specific physiological roles, such as direct toxicity and indirect influences on predator reproduction [8]. For example, in diatoms, unsaturated aldehydes 2E,4Z-deca-2,4-dienal and 2E,4Z,7Z-deca-2,4,7-trienal play a role in regulating the population dynamics of phytoplankton by reducing the viability of copepod eggs [9]. However, little is known about the role of volatile oxylipins in macroalgae, and it remains largely unexplored whether these volatile oxylipins are simply byproducts of metabolism or if they are actively produced for chemical defense purposes.

However, to the extent known the pathogen- and stress-induced cellular responses of marine algae are strikingly similar to those observed in animals and terrestrial plants, suggesting that the underlying biochemical pathways may have arisen early in evolutionary history [1]. Algae do not have a vascular system [10], indicating that a systemic response to attacks or stresses is not possible via internal signaling molecules. Therefore, akin to higher plant species, external chemical cues are necessary for communication within an alga, between individuals of a species, as well as with other organisms. It is conceptually attractive to propose that some marine algae may have evolved a capacity to recognize the identity of predatory or infectious organisms, thereby enabling adjustment of their chemical defenses accordingly. However, the mechanisms underlying communication between adjacent individuals of an alga species have generally been unclear, and there have been few reported signals mediating such interactions between marine plants. For example, Müller and Jaenicke found s serial of hydrocarbons as pheromones released from female gametes of brown algae can attract male gametes to form a diploid zygote [11]. In general, the low concentration of such molecules in the natural environment along with their relative chemical instability has presented considerable challenge in identifying molecules involved in allelopathic signaling in algae.

Previous studies have reported that *Pyropia haitanensis* can convert large quantities of C20:4 fatty acids into 1-octen-3-ol using a lipoxygenase (LOX) enzyme upon induction by agaro-oligosaccharides or high-temperature stress [12–14]. Such a general response suggests that 1-octen-3-ol may play an important role in response to both biotic and abiotic stress in *P. haitanensis*. The volatile 1-octen-3-ol is a fatty acid fragrant originally identified in fungi but has since been found in a wide variety of plants [15–17]. In nature, 1-octen-3-ol serves as a signaling molecule in plant cellular responses, plant-herbivore interactions, and plant-plant interactions. For example, in *Arabidopsis* 1-octen-3-ol induces expression of defense genes that are normally up-regulated by wounding or ethylene/jasmonic acid signaling. In addition, treatment with 1-octen-3-ol inhibits the expansion of necrotic lesions on *Arabidopsis* leaves [17]. As 1-octen-3-ol serves as a stress response molecule in terrestrial plants, it may conceivably serve one of the following roles in algae: (i) a direct effector on microorganisms infecting the thalli; (ii) an indirect communication molecule that serves to “prime” algae (‘alga-alga signaling’); or (iii) an inducer that initiates the defense response of plants. Moreover, relatively little is known about the ability of a volatile molecule to diffuse through an aqueous environment, amplify a signal and effectively achieve a physiological response.

The genus *Pyropia* has recently gained momentum as a model species for basic and applied studies in marine algal science [18]. In the present study, we aimed to investigate the role of 1-octen-3-ol in inter-algal signaling using *P. haitenansis*. Young thalli of *P. haitenansis* were challenged with 1-octen-3-ol, and the associated bacteria, redox state and volatile oxylipin biosynthetic pathways were monitored. Additionally, gene expressions and enzyme activities were also examined.

## Results

### Effect of 1-octen-3-ol on the decay rate and epiphytic bacteria of *P. haitanensis*

**Fig. 1:**
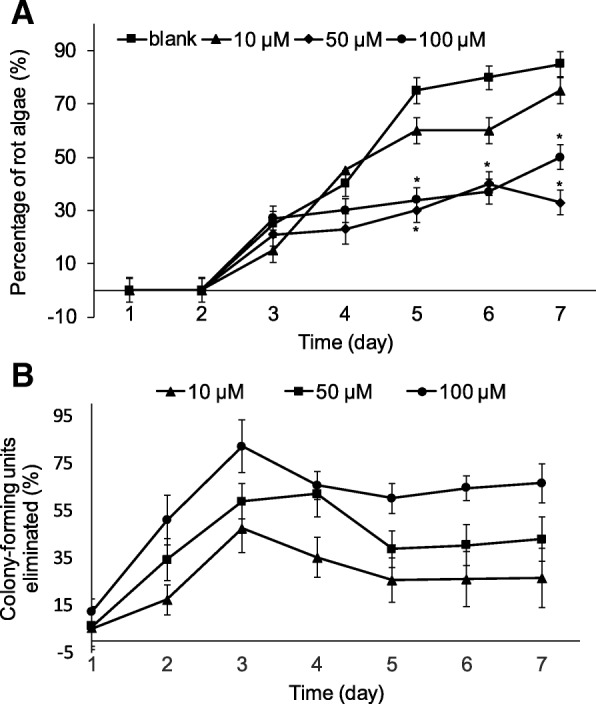
Effect of 1-octen-3-ol on decay rate and amount of bacteria associated with *P. haitanensis*. Blades (density of 7 mg/mL) were treated with different concentrations of 1-octen-3-ol for 7 days. **a**, The decay rate was recorded every day, as indicated by the amount of bleached surface area on a leaf. **b**, Bacteria associated with thalli were quantified by calculating the number of colony forming units on plates. The inhibition rate was calculated by comparing with the negative control. *n* = 10, ^*^P < 0.05, compared with control

First, we explored the effect of exogenously applied 1-octen-3-ol on the decay rate and quantity of epiphytic bacteria on thalli of *P. haitanensis*. Untreated control *P. haitanensis* thalli began to show signs of decay as evidenced by a bleached surface on day 3. The rate of decay in the control group further increased after day 4, and was significantly higher than that in the 1-octen-3-ol treatment groups (Fig. 1a). Indeed, the 1-octen-3-ol treatment groups showed a concentration-dependent reduction in thallus bleaching. Treatment with 10 μM of 1-octen-3-ol caused a moderate reduction in decay whereas a remarkable reduction was observed with either 50 or 100 μM of 1-octen-3-ol; however, there was no appreciable difference in decay reduction between the two higher concentrations. On day 7, the decay level of the 50 μM treatment group was significantly lower (2.6-fold) compared with the control group (*P* < 0.01), indicating that 50 μM of 1-octen-3-ol was sufficient to inhibit decay of *P. haitanensis* thalli.

Next, we examined the effect of applied 1-octen-3-ol on the quantity of epiphytic bacteria present on *P. haitanensis* thalli. Treatment with 1-octen-3-ol reduced the amount of epiphytic bacteria on *P. haitanensis* in a concentration-dependent manner. The greatest inhibitory effect was noted on day 3 at a treatment level of 100 μM 1-octen-3-ol (82.1% compared with the untreated control). However, the level of bacterial growth inhibition was attenuated upon prolonged culture, and eventually stabilized at 60% of control levels after 5 days (Fig. 1b).

### Redox state of *P. haitanensis* in response to 1-octen-3-ol application

**Fig. 2:**
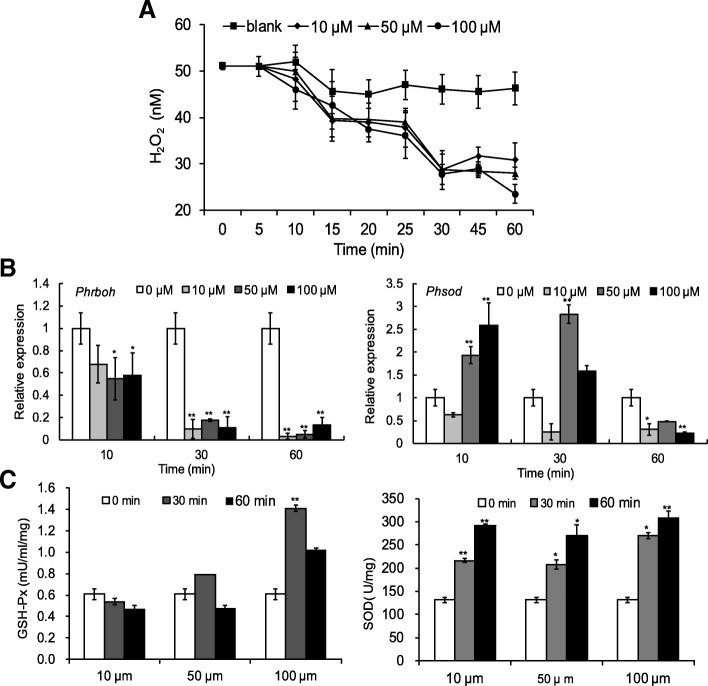
The redox state of *P. haitanensis* treated by 1-octen-3-ol. **a**, H_2_O_2_ concentration. Blades (density of 7 mg/mL) were treated with different concentrations of 1-octen-3-ol for 60 min, and the H_2_O_2_ concentration in the medium was measured at different time points. **b**, Relative expressions of *Phrboh* and *Phsod*. **c**, Antioxidant activities of SOD and GSH-Px. *n* = 3, ^*^P < 0.05, ^**^P < 0.01, compared with 0 μM treatment group

Thalli treated with various concentrations of 1-octen-3-ol were assessed for their redox state by measurement of H_2_O_2_, mRNA levels of two antioxidant genes *Phrboh* and *Phsod* (genes encoding NADPH oxidase and superoxide dismutase in *P. haitanensis*), and levels of two antioxidant enzymes, SOD and GSH-Px. As shown in Fig. 2a, 1-octen-3-ol exposure did not result in the release of H_2_O_2_ in *P. haitanensis*. In contrast, H_2_O_2_ concentrations were decreased compared with the control group, and continued to decrease over time. We noted an especially sharp decline at 25–30 min of 1-octen-3-ol exposure, and after 60 min, the H_2_O_2_ concentration in the 100 μM treatment group was decreased to 23.5 ± 2.1 μM, which was approximately two-fold lower compared with the control group (*P* < 0.01). However, there was no correlation between the levels of H_2_O_2_ and applied 1-octen-3-ol concentrations, which may signify that a maximal response was achieved at the lowest levels of 1-octen-3-ol exposure.

RT-PCR was utilized to examine the expressions of two genes, *Phrboh* and *Phsod*, which are related to the intracellular redox state of *P. haitanensis* thalli. After treatment with 1-octen-3-ol for 10 min, the expression of *Phrboh* was significantly decreased (*P <* 0.05), and was further reduced at 30 and 60 min with 10 μM of 1-octen-3-ol (*P <* 0.01), eventually showing a 30-fold decrease compared with the control group at 60 min (*P* < 0.01) (Fig. 2b). However, reduction in *Phrboh* by 1-octen-3-ol was not concentration-dependent. In contrast, *Phsod* expression was increased by 50 μM and 100 μM treatments of 1-octen-3-ol (*P* < 0.01) within the first 10 min of exposure. Maximal expression was observed at 10 min with 100 μM of 1-octen-3-ol; maximal expression was delayed to 30 min with application of 50 μM 1-octen-3-ol (2.8-fold, *P* < 0.01). At 60 min, *Phsod* expression was lower than that of the control group after all three concentrations of 1-octen-3-ol treatment (*P* < 0.01, Fig. 2b).

In addition, we evaluated the effects of exogenous application of 1-octen-3-ol on endogenous production of two of the primary antioxidant enzymes in cells, SOD and GSH-Px (Fig. 2c). The SOD activity was significantly increased after treatment of *P. haitanensis* with all three concentrations of 1-octen-3-ol, and it was further increased in a time-dependent manner up to 60 min (Fig. 2c). However, the GSH-Px activity did not respond as strongly to exogenous application of 1-octen-3-ol, and only 100 μM of 1-octen-3-ol increased its activity at 30 and 60 min (*P* < 0.01).

### Lipidomic analyses of *P. haitanensis*

**Fig. 3:**
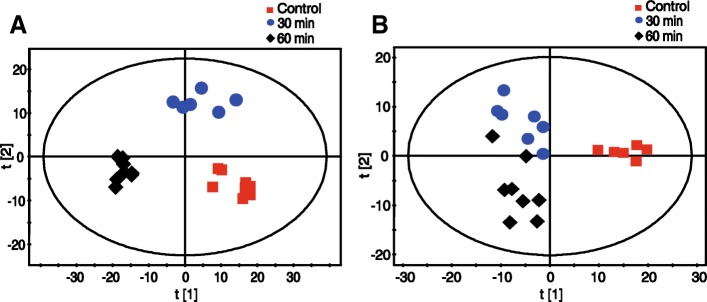
PLS-DA cross-validated score plot for *P. haitanensis* extracts cultured under control and 1-octen-3-ol-treated conditions. **a**, Positive; **b**, Negative. *n* = 8

Changes in the lipid profile of *P. haitanensis* in response to application of 50 μM of 1-octen-3-ol were characterized by UPLC-Q-TOF-MS and multivariate statistical analysis. Additional file 1: Figure S1 illustrates the LC-MS chromatograms in positive and negative modes. A total of 8110 signals were detected in the ESI^+^ mode, and 7712 signals were detected in the ESI^−^ mode. The PCA score plots indicated that *P. haitanensis* underwent lipidomic changes after exposure to 1-octen-3-ol (Additional file 2: Figure S2). Partial least squares-discriminant analysis (PLS-DA) revealed a clearer segregation between the control group and the two 1-octen-3-ol treated groups, indicating that the lipid profile continued to change from 30 to 60 min (Fig. 3). There were 101 up-regulated compounds and 196 down-regulated compounds in *P. haitanensis* after 30 min, while 131 and 268 compounds were increased and decreased after 60 min, respectively. Based on online lipid databases and fragmentation pathways, 25 significantly altered lipid biomarkers (*P* < 0.05) were identified (Additional file 3). The majority of these biomarkers were membrane lipids (phospholipid and glycolipid), including phosphatidylcholine (PC), phosphatidic acid (PA), phosphatidylglycerol (PG), phosphatidylinositol (PI), Lyso-PC, Lyso-PG, Lyso-PI, Lyso-PA, Lyso-phosphatidylethanolamine (PE), Lyso-digalactosyldiacylglycerol (DGDG) and Lyso-sulfoquinovosyldiacylglycerol (SQDG) (Additional file 4: Table S1).

**Fig. 4:**
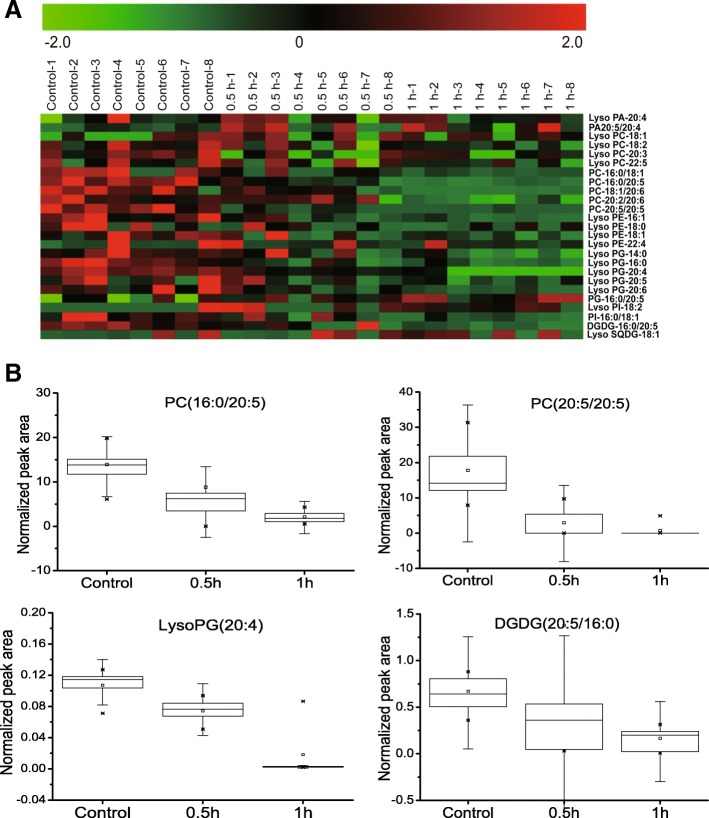
**a**, Heatmap of significantly different lipid levels in response to 1-octen-3-ol treatment. Red indicates high levels of lipids, whereas green indicates low levels. The axis represents the samples as three groups (control vs. 50 μM of 1-octen-3-ol treatment for 0.5 h or 1 h). The vertical axis represents the variations between the 25 different lipids. **b**, Boxplot visualizations of the relative abundances of significantly changed metabolites in the control and samples treated with 1-octen-3-ol. The box and whisker plots represent the maximum and minimum values (whiskers), the upper and lower quartiles (boxes), and the median (middle horizontal line)

The heatmap of these lipid levels revealed changes in the *P. haitanensis* lipidome after exposure to 1-octen-3-ol: red indicates high levels of lipids, whereas green indicates low levels (Fig. 4a). A large number of membrane lipids in *P. haitanensis* were decreased with treatment of 1-octen-3-ol, including PC (20:5/20:5), PC (16:0/18:1), PC (18:1/20:6), PC (16:0/20:5), Lyso PG (20:4), LysoPG (20:6) and DGDG (20:5/16:0). Additionally, a variety of lipids also decreased over time in these exposure experiments, and were mostly those that contained unsaturated fatty acids, such as PC and DGDG containing C20:5 or Lyso PG containing C20:4. The content of PC (20:5/20:5), PC (16:0/20:5), Lyso PG (20:4) and DGDG (20:5/16:0) in *P. haitanensis* were decreased by 4.6-, 2.7-, 2.1- and 2.0-fold, respectively, after treatment with 1-octen-3-ol for 60 min. Further boxplot visualization analysis of the data also revealed the significant decrease of these four membrane lipids (Fig. 4b).

### Effects of 1-octen-3-ol on phospholipase 2 activity

**Fig. 5:**
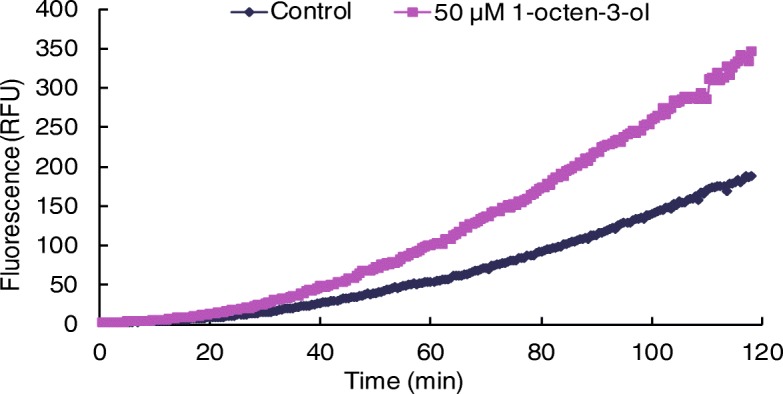
Effects of 1-octen-3-ol on PLA_2_ activity in *P. haitanensis*

The effect of 50 μM of 1-octen-3-ol on PLA_2_ activity was next tested in *P. haitanensis* thalli. PED6 was used as the fluorogenic substrate for PLA_2_. Both the control and 1-octen-3-ol-treated groups displayed a continuous increase in the fluorescence signal; however, 1-octen-3-ol treatment increased the rate of fluorescence apparently compared with the control group, suggesting an activation of PLA_2_ (Fig. 5).

### Effects of 1-octen-3-ol on the free fatty acid (FFA) profile of *P. haitanensis* thalli

Several saturated and unsaturated FFAs were detected in *P. haitanensis* thalli by GC-MS (Table 1). Among these FFAs, the concentrations of palmitic acid (C16:0), arachidonic acid (C20:4) and eicosapentaenoic acid (C20:5) were high, accounting for 83.5% of total FFAs. Treatment with 1-octen-3-ol increased the total amount of FFAs in *P. haitanensis*. In particular, C20:1, C20:4 and C20:5 were significantly increased after 30 min of treatment with 50 μM of 1-octen-3-ol (*P* < 0.05) whereas C18:0 was dramatically increased in the 10 μM treatment group (*P* < 0.01). In contrast, most of the FFAs were decreased after 60-min treatment compared with the 30-min treatment at the same concentration. For example, C20:4 concentration at 60 min was 59.54% of that at 30 min (*P* < 0.01), while the concentration C20:5 was lower than that of the untreated control. The concentration of C18:3 in *P. haitanensis* was very low in control thalli, and it became undetectable after 1-octen-3-ol treatment. No consistent trends were noted for other FFAs in *P. haitanensis* when treated with 1-octen-3-ol.

**Table 1:** Effects of 1-octen-3-ol on the levels of FFAs in *P. haitanensis* (μg/g)

Fatty acids	Control	30 min			60 min		
		10 μM	50 μM	100 μM	10 μM	50 μM	100 μM
C14:0	10.90 ± 5.77	47.72 ± 5.40	17.05 ± 3.86	23.94 ± 12.30^*^	20.47 ± 4.10	9.70 ± 3.14^#^	10.17 ± 1.12
C16:0	91.32 ± 12.56	96.65 ± 1.71	106.13 ± 15.49	108.91 ± 10.17	89.98 ± 9.64	89.98 ± 5.71	99.71 ± 5.41
C18:0	28.53 ± 2.27	37.95 ± 0.22^**^	32.28 ± 5.61	32.21 ± 1.18	25.91 ± 7.03^#^	29.25 ± 5.10	25.20 ± 4.85
C18:1	2.64 ± 0.54	3.51 ± 0.24	3.67 ± 0.66	3.18 ± 0.42	2.48 ± 0.99	2.29 ± 0.57	2.82 ± 0.18
C18:2	1.39 ± 0.24	1.67 ± 0.21	2.00 ± 0.47	1.74 ± 0.26	0.96 ± 0.29	1.22 ± 0.51	1.61 ± 0.09
C18:3	0.20 ± 0.08	–	–	–	–	–	–
C20:1	2.63 ± 0.19	2.92 ± 0.28	5.78 ± 1.58^*^	4.48 ± 1.21	2.73 ± 0.75	3.03 ± 0.81	3.56 ± 0.18
C20:2	1.96 ± 0.24	1.75 ± 0.10	3.40 ± 1.09	2.40 ± 0.29	2.95 ± 1.25	2.27 ± 0.21	1.93 ± 0.01
C20:4	41.03 ± 17.17	42.36 ± 9.39	64.32 ± 9.11^*^	48.64 ± 4.00	39.50 ± 4.07	38.30 ± 3.14^##^	44.88 ± 1.76
C20:5	109.19 ± 22.94	114.54 ± 8.10	140.56 ± 12.35^*^	126.12 ± 7.93	96.48 ± 6.63	105.01 ± 10.34^#^	123.23 ± 4.66

^*^P < 0.05; ^**^P < 0.01, compared with control, *n* = 3; ^#^*P <* 0.05; ^##^*P <* 0.01, compared with the same concentration groups after 30 min of treatment. “-” indicates undetected

### Lipoxygenase gene expression by *P. haitanensis* in response to 1-octen-3-ol treatment

**Fig. 6:**
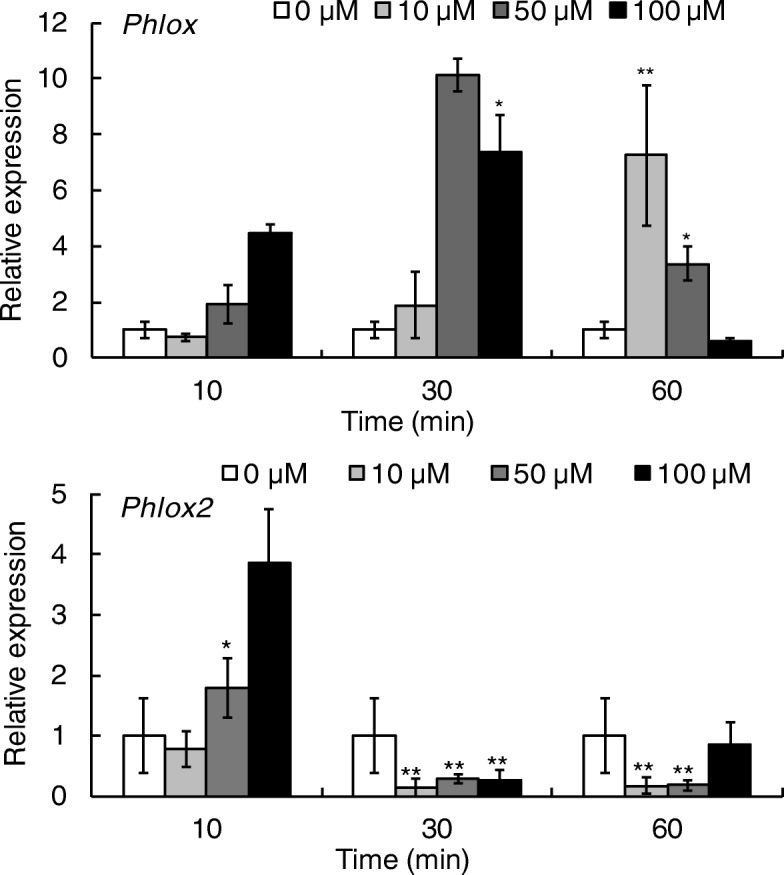
Relative expressions of *Phlox* and *Phlox2* in *P. haitanensis* after treatment with 1-octen-3-ol. *n* = 3, ^*^P < 0.05, ^**^P < 0.01, compared with 0 μM treatment group

We also assessed the relative expressions of two LOX-encoding genes, *Phlox*, and *Phlox2*, which may be involved in oxylipin biosynthesis, in response to 1-octen-3-ol exposure (Fig. 6). Treatment with 10 μM 1-octen-3-ol triggered a time-dependent increase in the *Phlox* expression. In the 50 μM and 100 μM treatment groups, an increase in expression at both 10 and 30 min was observed. In the 50 μM treatment, *Phlox* expression was increased by over 10-fold at 30 min (*P* < 0.01). Expression of *Phlox2* in *P. haitanensis* was increased after 1-octen-3-ol treatment for 10 min; however, after 30 min it was dramatically decreased (*P* < 0.01).

### Changes of oxylipins after 1-octen-3-ol treatment

Oxylipins are the downstream oxidative products of polyunsaturated fatty acids (PUFAs). A few C18-derived oxylipins were detected in untreated *P. haitanensis*, such as 9-hydroperoxy octadecadienoic acid (HpODE) and 13-hydroperoxy octadecatrienoic acid (HpOTE), which are commonly observed in higher plants. However, some common C20-derived oxylipins were not detected, such as 12-hydroperoxy eicosatetraenoic acid (HpETE) and 12-hydroperoxy eicosapentaenoic acid (HpEPE). Moreover, several ketol compounds, including 9,10-α-ketol and 9,12-γ-ketol, and several hydroxylated oxylipins, such as 9-hydroxy octadecatrienoic acid (HOTE), 8-hydroxy eicosatetraenoic acid (HETE) and 8-hydroxy eicosapentaenoic acid (HEPE), were detected. After 1-octen-3-ol treatment for 30 min, most of the C18 oxylipins were significantly decreased. For example, 9-HpODE, 9-HpOTE and 13-HpOTE were markedly decreased after treated with 50 μM of 1-octen-3-ol (*P* < 0.01). In contrast, the C20 derivatives were significantly increased at 30 min, especially several C20:5-derived oxylipins, such as 8-HpEPE, 9,12-γ-ketol and 8-HEPE (*P* < 0.01). However, there was a large decrease of these derivatives in the 10 μM and 50 μM treatment groups at 60 min, such as 8-HEPE (*P* < 0.01, Table 2, Additional file 5: Figure S3).

**Table 2:** Changes in oxylipin contents from *P. haitanensis* treated with 1-octen-3-ol. Data were represented by integrated peak area (×10^7^)

Precursor fatty acids	Oxylipins	Control	30 min			60 min		
			10 μM	50 μM	100 μM	10 μM	50 μM	100 μM
C18:2	9-HpODE	131.94 ± 2.23	95.72 ± 15.52	31.45 ± 6.27^**^	88.2 ± 8.28	90.7 ± 16.69	64.63 ± 7.24^*^	48.04 ± 3.36^*^
	9,10-α-ketol	157.17 ± 1.68	90.64 ± 12.32	24.7 ± 4.23^**^	95.14 ± 15.24	97.63 ± 12.16^*^	66.19 ± 8.26^*#^	45.23 ± 3.56^*^
ω3-C18:3	13-HpOTE	4.27 ± 0.31	3.11 ± 0.43	1.45 ± 0.35^*^	2.28 ± 0.24	2.65 ± 0.36^**^	2.23 ± 0.27^*^	1.64 ± 0.54^*^
	9-HpOTE	31.98 ± 3.1	20.19 ± 3.24	4.54 ± 0.52^**^	16.57 ± 1.68	20.1 ± 2.05^**^	14.66 ± 4.76^*^	7.24 ± 1.21^**^
	9,10-α-ketol	88.53 ± 0.89	55.01 ± 8.67	14.29 ± 3.25^**^	57.91 ± 12.24	69.99 ± 12.24	43.9 ± 6.28^*^	26.97 ± 4.24^*^
γ-C18:3	9-HpOTE	3.34 ± 0.37	2.48 ± 0.32	1.12 ± 0.32^**^	0.87 ± 0.24^*^	1.48 ± 0.35^*^	1.71 ± 0.37^*^	0.73 ± 0.13^**^
	13-HpOTE	7.8 ± 0.14	6.71 ± 0.42	5.57 ± 0.56	4.54 ± 0.68^*^	5.7 ± 1.37	5.27 ± 0.17	4.57 ± 1.41
	9-HOTE	7.28 ± 0.14	4.87 ± 0.54	3.35 ± 0.68^*^	5.07 ± 1.14	4.49 ± 0.77^*^	3.59 ± 0.27^**^	4.49 ± 0.56
C20:4	9,12-γ-ketol	24.59 ± 2.87	22.59 ± 1.1	28.82 ± 4.24	21.37 ± 3.11	17.66 ± 0.99^*^	20.48 ± 2.05	21.65 ± 1.92
	8-HpETE	–	0.94 ± 0.15	4.34 ± 1.24	3.00 ± 0.54	2.75 ± 0.37^**^	3.89 ± 0.86	5.23 ± 1.02
	8-HETE	11.59 ± 0.61	13.11 ± 2.65	26.99 ± 5.42^*^	15.09 ± 3.28	6.19 ± 1.73^*^	7.36 ± 1.25^#^	20.45 ± 4.04
C20:5	8-HpEPE	8.83 ± 0.59	20.17 ± 1.98	52.29 ± 12.24^**^	19.73 ± 5.24	5.52 ± 0.43	19.87 ± 3.26^#^	27.08 ± 4.25
	9,12-γ-ketol	13.32 ± 0.89	20.54 ± 5.21	54.27 ± 10.25^**^	25.07 ± 5.44	13.14 ± 2.34	22.51 ± 5.23	35.57 ± 5.23
	8-HEPE	30.54 ± 4.86	44.67 ± 8.56	102.38 ± 20.24^**^	54.34 ± 8.68	20.07 ± 4.45^*^	35.62 ± 7.34^##^	68.43 ± 6.23

^*^P < 0.05; ^**^P < 0.01, compared with control, n = 3; ^#^*P <* 0.05; ^##^*P <* 0.01, compared with the same concentration groups after 30 min of treatment. “-” indicates undetected

### Induction of volatile oxylipins by 1-octen-3-ol

GC-MS was used to measure released volatile oxylipins in response to 1-octen-3-ol treatment. A diversity of volatile oxylipins were detected in *P. haitanensis*, including short-chain unsaturated alcohols, aldehydes and ketones. C8 derivatives were the most diverse, while 1-octen-3-one and 2-octenal were the most abundant. The concentrations of all volatiles were increased in the 10 μM and 50 μM treatment groups after 1-octen-3-ol treatment, and such increase was maintained up to 60 min. Several significantly increased volatiles included 1-octen-3-ol, 1,5-octadiene-3-ol, 2,6-nonadienal, 1-penten-3-one and 2,4-heptadienal (*P* < 0.05). Especially, 1-octen-3-ol itself was markedly increased when *P. haitanensis* was treated with 50 μM of 1-octen-3-ol (*P* < 0.01). However, no significant increase of volatiles was found when treated with 100 μM of 1-octen-3-ol. Instead, the concentrations of some volatiles were decreased over those of the control group (Table 3).

**Table 3:** Changes in volatile oxylipin contents from *P. haitanensis* after 1-octen-3-ol treatment. (μmol/g)

Volatiles	Control	30 min			60 min			
		10 μM	50 μM	100 μM	10 μM	50 μM	I^a^ + 50 μM	100 μM
2-pentenal	1.98 ± 0.18	2.17 ± 0.22	2.45 ± 0.37	2.11 ± 0.34	2.25 ± 0.31^*^	2.59 ± 0.18	1.97 ± 0.22^#^	1.93 ± 0.32
2,4-pentadienal	0.30 ± 0.06	0.74 ± 0.17	0.71 ± 0.15	0.64 ± 0.16	1.28 ± 0.54	4.45 ± 0.05^**^	3.96 ± 0.28	0.57 ± 0.15
1-penten-3-one	2.34 ± 0.30	2.41 ± 0.32	4.07 ± 0.38^*^	3.02 ± 0.50	3.26 ± 0.37	5.72 ± 0.32^*^	4.01 ± 0.29	2.68 ± 0.26
2,4-heptadienal	0.19 ± 0.05	0.31 ± 0.02	0.82 ± 0.15^*^	0.28 ± 0.03	0.39 ± 0.02^*^	1.63 ± 0.06^**^	1.44 ± 0.24	0.30 ± 0.05
2-octen-1-ol	1.32 ± 0.16	1.71 ± 0.31	1.81 ± 0.90	1.38 ± 0.36	2.12 ± 0.40	2.25 ± 0.32	1.55 ± 0.20^#^	1.81 ± 0.01^*^
1-octen-3-ol	1.68 ± 0.14	2.03 ± 0.25	5.82 ± 0.41^**^	1.62 ± 0.51	3.01 ± 0.24^*^	6.25 ± 0.20^**^	2.02 ± 0.17^##^	2.36 ± 0.27^*^
1,5-octadiene-3-ol	2.60 ± 0.15	4.32 ± 0.27^*^	9.63 ± 0.45^**^	2.49 ± 0.87	4.28 ± 0.05^**^	6.08 ± 0.06^**^	3.77 ± 0.17	3.30 ± 0.35^*^
1-octen-3-one	58.96 ± 5.75	59.02 ± 0.23	61.38 ± 0.34	59.68 ± 0.26	59.48 ± 0.26	60.89 ± 3.00	61.91 ± 2.58	58.04 ± 0.36
2-octenal	19.42 ± 0.28	19.68 ± 0.22	21.71 ± 0.19	20.05 ± 0.11	20.52 ± 0.29	23.11 ± 0.44	20.72 ± 0.43	19.65 ± 0.41
2,6-nonadienal	0.17 ± 0.05	0.34 ± 0.03	0.81 ± 0.14^*^	0.22 ± 0.44	0.36 ± 0.14	1.39.6 ± 0.07^**^	0.98 ± 0.06^#^	0.19 ± 0.06

^*^P < 0.05; ^**^P < 0.01, compared with control; ^#^*P <* 0.05; ^##^*P <* 0.01 compared with 60 min-50 μM, *n* = 3. “-” indicates undetected

^a^“I” is n-propylgallate, 60 μM, a non-selective inhibitor of LOXs

When thalli were pretreated for 60 min with 60 μM n-propyl gallate, a non-selective inhibitor of LOXs [19, 20] and then treated with 50 μM 1-octen-3-ol for 60 min, the concentrations of many volatiles were decreased over 1-octen-3-ol only treated thalli, including 1-octen-3-ol, 2-octen-1-ol, 2-pentenal, 2-octenal and 2,6-nonadienal (*P* < 0.05). Taken together, these finding strongly suggested that the production of these volatiles was dependent on LOX activity.

### Changes of phytohormones after 1-octen-3-ol treatment

**Fig. 7:**
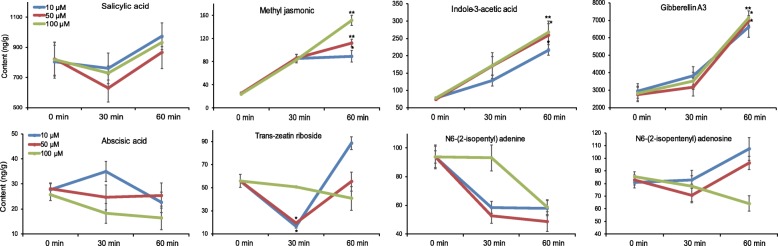
Phytohormones in *P. haitanensis* after exposure to 1-octen-3-ol. The thalli treated with different concentrations of 1-octen-3-ol for different time periods were extracted and analyzed by LC-MS. The quantities of phytohormones were determined using standard curves. ^*^P < 0.05, ^**^P < 0.01, compared with 0 min groups

Volatile oxylipins have been implicated to modulate plant defense and development via phytohormone signaling. Some hormones, such as jasmonic acid, are in fact downstream metabolites of oxylipin metabolism [21]. Therefore, we measured phytohormone levels in *P. haitanensis* by LC-MS to assess the effect of 1-octen-3-ol on this process. Eight phytohormones were detected, including methyl jasmonic acid (MeJA), gibberellin A3 (GA3), indole-3-acetic acid (IAA), salicylic acid, abscisic acid, trans-zeatin riboside, brassinolide, N6-(2-isopentyl) adenine and N6-(2-isopentenyl) adenosine (Fig. 7, Additional file 6: Figure S4). After 1-octen-3-ol treatment, the concentrations of three phytohormones (IAA, MeJA and GA3) were increased in a time-dependent manner. The content of MeJA in *P. haitanensis* was increased by ~ 3.4-fold after treatment with 100 μM of 1-octen-3-ol for 30 min, and it continued to increase up to 60 min. MeJA levels also increased in a concentration-dependent manner. IAA was consistently increased in both a time- and concentration-dependent manner after treatment, achieving a 3.4-fold increase compared with the control group at 100 μM of 1-octen-3-ol for 60 min (*P* < 0.01). There were no obvious changes in the concentrations of the other measured phytohormones.

## Discussion

In higher plants, short-chain oxylipins consisting of six to nine carbon atoms are often important signaling molecules and play physiological roles in (a)biotic defense mechanisms, such as wounding, pest attack, high light stress and water deficit [21, 22]. The presence of volatile oxylipins has also been reported in several marine diatoms [9, 11] and macroalgae [6–8]. However, the functions of these molecules in macroalgae have not been fully investigated, although their potential role as feeding attractants have been suggested in the green alga *Ulva pertusa* [23]. In addition, 4-HHE, a C6-hydroxylated aldehyde in *Laminaria digitata*, was reported to induce modifications in the oxylipin profile of this brown alga [24]. Therefore, it remains relatively unexplored whether algae can use lipophilic oxylipins to signal needed stress responses to members of the same species. Preliminary studies have demonstrated that *P. haitanensis* cells can convert C20:4 to 1-octen-3-ol via lipoxygenase when confronted with modifications in their environment [13, 14], and algae may use released 1-octen-3-ol as a chemical signal as shown in the present work.

Treatment with 1-octen-3-ol reduced the amount of epiphytic bacteria present on *P. haitanensis* blades, indicating that 1-octen-3-ol might be an elicitor that induces *P. haitanensis* resistance. Oxidative burst is a common and primary mechanism by which algae respond to external stimuli or pathogens, often leading to release of transient bursts of H_2_O_2_ [25]. It has been reported that 1-octen-3-ol also induces an oxidative burst (e.g., H_2_O_2_) in *Arabidopsis thaliana* leaves [17]. Surprisingly, 1-octen-3-ol exposure did not trigger an oxidative burst in *P. haitanensis*. On the contrary, 1-octen-3-ol treatment resulted in a dramatic reduction of H_2_O_2_. In addition, the expression of genes encoding enzymes associated with intracellular redox state were found to have similar changes. NADPH oxidase is an enzymatic source of cellular reactive oxygen species (ROS), generating superoxide anions by transferring electrons from intracellular NADPH and reducing molecular oxygen [25]. NADPH oxidase is activated when some algae are stimulated by external factors, such as oligosaccharides or lipopolysaccharides [26]. But here, *Phrboh* expression was down-regulated in response to 1-octen-3-ol exposure, which was consistent with the decreased H_2_O_2_ concentration, especially after 30 min of treatment. These results indicated that 1-octen-3-ol exposure did not cause intracellular ROS generation, but rather, inhibited ROS synthesis. SOD is an important antioxidant in cellular systems. The expression of *Phsod*, on the other hand, was up-regulated after the early phase of stimulation, and SOD activity was increased correspondingly, suggesting that 1-octen-3-ol stimulated cellular antioxidant mechanisms. However, 1-octen-3-ol exposure appears to stimulate only a burst of mRNA transcription related to antioxidants. After 60 min of exposure *Phsod* expression was decreased whereas SOD activity remained constant. Furthermore, the enzyme activity of intracellular GSH-Px, an antioxidant associated with H_2_O_2_ metabolism, was also increased after treatment with 1-octen-3-ol. This finding suggested that the large decrease in H_2_O_2_ observed in this study could be attributed to activation of an antioxidant system in response to 1-octen-3-ol, indicating that this molecule plays a different role in algae compared with higher plants. Furthermore, the algae’s response to 1-octen-3-ol was different from other previously studied stimulants [13, 27–29]. Therefore, the reduction of epiphytic bacterial on *P. haitanensis* could not be attributed to an oxidative burst.

Marine algae can be warned of predators or pathogens by their conspecific neighbors [6]. Algal communication using volatile oxylipins as messengers requires relatively large concentrations in order to reach neighboring algae or even distal parts of the emitter, because of substantial dilution during water transport. Therefore, a signal transduction system is likely required to amplify the signal. Upon treatment with 1-octen-3-ol, *P. haitanensis* upregulates a number of pathways at both the transcriptional and metabolic levels. Synthesis of volatile oxylipins is initiated by the concerted action of lipases and phospholipases through deacylation of glycolipids or phospholipids to release FFAs. PUFAs are then converted by LOXs into PUFA hydroperoxides, such as 13-HpOTE and 9-HpODE. Subsequently, fatty acid hydroperoxide lyase (HPL) cleaves fatty acid hydroperoxides to form short-chain aldehydes, which can be further converted to ketones or alcohols [3, 4, 21, 22]. In *P. haitanensis*, the contents of C16:0, C20:4 and C20:5 fatty acids are relatively high. The lipid profile of *P. haitenansis* was previously investigated, and revealed that eicosapentaenoic acids are enriched at both the sn-1 and sn-2 positions of various phospholipids and glycolipids, including PA, PC, PG and DGDG [30]. In the current study, a large number of PUFA-enriched membrane esters were observed, such as PC (20:5/20:5), PC (16:0/20:5), Lyso PG (20:4) and DGDG (20:5/16:0). Moreover, a large number of these membrane lipids were decreased after 1-octen-3-ol treatment and further down-regulated over time, while PLA_2_ activity was increased. These findings suggested that PLA_2_ or other lipases might cleave phospholipids or glycolipids, resulting in the preferential release of PUFAs, such as C20:4 or C20:5. An increase in C20:4 and C20:5 FFAs was observed after 1-octen-3-ol treatment, indicating a specific increase in PUFAs upon 1-octen-3-ol treatment. Other studies have also reported that stimulation of algal tissue results in increased levels of free unsaturated fatty acids. For example, *L. digitata* releases FFAs when stimulated with lipopolysaccharides [24], while *Gracilaria chilensis* releases FFAs during wounding [31]. However, FFA levels were significantly reduced after 60 min of 1-octen-3-ol treatment, and C18:3 became undetectable after 1-octen-3-ol treatment. Similar results have not been reported in other algae.

Subsequently, these FFAs are transformed into a suite of other metabolites. Our lipidomic analysis revealed that 14 oxylipin compounds were produced and mainly derived from C18 and C20 unsaturated fatty acids, including hydroperoxy and hydroxy products as well as several ketols. Biosynthesis of oxylipins substantially involves LOXs and several members of the cytochrome P450 family (designated CYP74), which includes allene oxide synthase, HPL and divinyl ether synthase [8]. Several LOXs have been identified in *P. haitanensis*, such as Phlox and Phlox2 [12, 32], but there is no clear evidence of CYP74 enzymes in seaweeds. However, these two LOX enzymes are both multifunctional enzymes. Phlox has high HPL, LOX and allene oxide synthase activities within one catalytic domain of the protein, while Phlox2 can catalyze PUFAs to form a large amount of hydroperoxides and hydroxy derivatives [12, 32]. Therefore, these two lipoxygenases in *P. haitanensis* could be responsible for catalyzing the synthesis of the various products detected in this study. Moreover, we observed 1-octen-3-ol dramatically increased *Phlox* expression. In some PUFA-derived oxylipins, C20-derived products were significantly increased after 30 min of 1-octen-3-ol treatment, indicating that C20 fatty acids were converted to oxylipins in a short period of time by LOX, while, C18-derived oxylipins were significantly decreased after 30 min of treatment, but began to accumulate at 60 min, in contrast, C20-derived oxylipins were decreased at 60 min, indicating a shift in metabolism. Previous studies on Phlox and Phlox2 activities have revealed a preference of them for C20 fatty acids, catalyzing faster reaction rates compared with C18 fatty acids. As a result, C20-derived oxylipins are formed faster than C18-derived ones, which could explain the results observed in this study. However, what is the reason for the decrease of C18-derived oxylipins in a short period of time and C20-derived ones in the later stage? For example, hydroperoxy products derived from C18 fatty acids, such as 9-HpODE, 9-HpOTE and 13-HpOTE were significantly reduced after 1-octen-3-ol treatment for 30 min (*P* < 0.01). Moreover, very few hydroperoxy products (only 8-HpETE and 8-HpEPE) derived from C20 were detected in this study, while products that are common in algal species, such as 12-HpETE and 12-HpEPE, were not detected. It has been revealed that C20 PUFAs are plentiful in red algae, and oxylipins deriving from C20 PUFAs are also abundant [8]. Then, it remains unclear whether they are relatively rare in *P. haitanensis*, or if they are simply rapidly metabolized into other products? Our previous study found that Phlox has high HPL activity, especially for eicosapentaenoic acids, which can be rapidly transformed into unsaturated aldehydes and alcohols. For example, 12-HpETE can be completely metabolized to corresponding products within 6 s [12]. This result suggests that one reason why no hydroperoxy products were observed after 30 min in this study was the result of their rapid metabolism into volatile oxylipins. In the present study, 1-octen-3-ol treatment did cause the production of a broad range of short-chain volatile substances derived from oxylipins, such as 2-pentenal and 1-penten-3-one derived from the cleavage products of 13-HpOTE, 2-octen-1-ol and 1-octen-3-one derived from the cleavage products of 12-HpETE, and 2,6-nonadienal derived from 12-HpEPE [12]. In contrast to higher plants that use C18 fatty acids for the production of C6 volatile aldehydes, red algae generate C8 volatiles because C20 fatty acids are the main FFAs [3, 8].

We found that the contents of many volatiles, such as 2-octen-1-ol, 1-octen-3-ol, 2,6-nonadienal and 2-pentenal, were all significantly decreased when treated with n-propyl gallate, a well known LOX inhibitor. This result strongly suggests that their production depended upon metabolism of fatty acids by lipoxygenases and hydroperoxide lyases. Interestingly, the concentration of 1-octen-3-ol was significantly increased after treatment with 50 μM of 1-octen-3-ol, indicating that *P. haitanensis* might use a rapid autocatalytic synthetic cycle to amplify the signal for rapid transmission. Furthermore, these short-chain volatile oxylipins in *P. haitanensis* might also play a role in chemical defense, as 1-octen-3-ol treatment inhibited the growth of associated bacteria and reduced the decay of the *P. haitanensis* blade. Kim et al. have reported that 1-octen-3-ol and 2,4-heptadienal can strongly inhibit microorganism growth [33], and 2,6-nonadienal also demonstrates apparent bactericidal activity against many pathogens [34].

Volatile oxylipins can crosstalk with phytohormones, mostly JA, by influencing JA biosynthesis genes and JA-dependent signaling in several plant species [4, 35]. For example, pre-treatment of *Arabidopsis* plants with 2(*E*)-hexenal increases susceptibility to *Pseudomonas syringae* pv. *tomato* by activating the JA-dependent signaling pathway [36]. Exposure of *A. thaliana* to 1-octen-3-ol induces the expressions of defense genes that are associated with JA signaling and inhibits the growth of the pathogen *Botrytis cinerea* on infected leaves [17]. As the JA and MeJA are important activators of the immune response against insect herbivores, they may serve as warning mechanisms for plants against impending insect attacks, thus allowing them to induce or prime their defense mechanisms in systemic tissue as well as in neighboring plants [37]. Until recently, there is no conclusive evidence that JA and MeJA act as endogenous phytohormones in *Pyropia* species. Mikami et al. have reported that JA cannot be detected in *Pyropia yezoensis* [38], and this is consistent with findings wherein we also did not detect JA in the present study and under normal conditions of *P. haitanensis* [39]. However, when stimulated by mechanical damage or special elicitor (e.g., flg22), a small amount of JA can be detected [40, 41]. So the role of JA in red algae needs to be further examined. But we conformed that MeJA and other hormones were detected. Similar to land plants, 1-octen-3-ol treatment led to MeJA synthesis in *P. haitanensis*, as well as elevated levels of IAA and GA3. IAA is the most crucial natural auxin in plants, playing a central role in cell division, elongation and development [42], while gibberellins have a number of effects on plant development, stimulating rapid growth of stem and root, inducing mitotic division in the leaves of some plants, and increasing seed germination rates [43]. Presently, it is not clear whether MeJA and other phytohormones can serve similar roles in algae as they do in terrestrial ecosystems. The current works suggests that they may be involved in inducing algal defenses and in triggering a “priming” state, preparing the alga to respond to anticipated stressors or pathogen attack. It has been reported that addition of MeJA to *Chondrus crispus* induces increased enzyme activities that are potentially involved in defensive reactions [44]. Previously, we have observed that 1-octen-3-ol enhances primary metabolism in *P. haitanensis* and promotes cell growth [45]. Herein, the induction of the antioxidant system, as well as increased defense and growth-related hormones, suggests that 1-octen-3-ol might prime *P. haitanensis* to prepare for an impending stress or pathogen attack.

## Conclusion

**Fig. 8:**
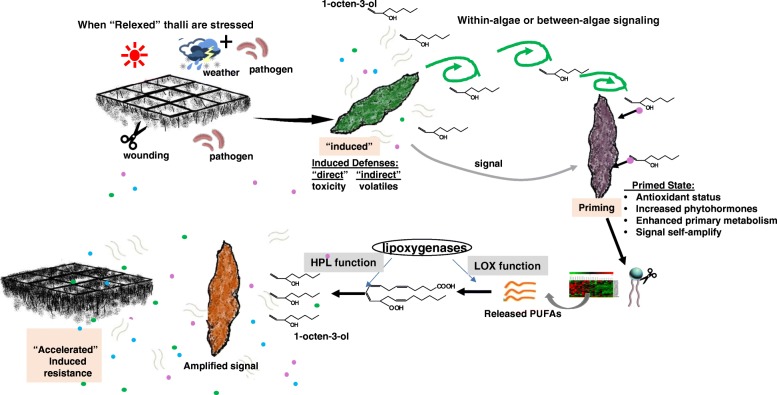
A hypothetical model of 1-octen-3-ol signal transduction and amplification among *P. haitanensis*

In response to external stimuli, rapidly produced 1-octen-3-ol self-amplifies via a positive feedback of the fatty acid-oxylipin metabolic cycle. This loop allows for continuous production, thereby providing a rapid, reliable and highly mobile signal that could transfer messages to neighbors or distal thalli at relatively high concentrations. Moreover, 1-octen-3-ol may transform *P. haitenansis* to a “primed” state, capable of rapid upregulation of its defense machinery by enhancing the synthesis of MeJA, IAA and GA3, adjusting the cellular redox state and promoting primary metabolism and cell growth. This ultimately leads to activation of the defense response (Fig. 8).

## Materials and methods

### Materials

The thalli of Zhedong-1 (a variety of cultivars) were collected from the coast of Xiangshan harbor in Zhejiang province, China, (longitude 121.56.153, latitude 29.05.065) in October 2016. Sampling was permitted by the local government (Xiangshan County Government) and the local department of fisheries (Ningbo Ocean & Fishery Bureau). *P. haitanensis* thalli that were 2–10 cm in length were collected and dehydrated at room temperature in the shade before being stored at − 20 °C.

### Cultivation and treatments of samples

The samples were rinsed with filtered seawater and then cleaned with 0.7% KI (*w*/*v*) for 10 min. Selected healthy thalli samples were rehydrated in sterilized seawater at 20 °C for 24–48 h under 40–50 μmol photons m^− 2^ s^− 1^ (with a photoperiod of 12:12 h) before use. For the 1-octen-3-ol stimulation study, 1-octen-3-ol (Sigma-Aldrich Inc., St. Louis, MO, USA, enantiomeric mixture) was dissolved in ethanol. The thalli were grown in seawater at a density of 7 mg/mL, and then exposed to 1-octen-3-ol at final concentrations of 10, 50 and 100 μM at 20 °C for 30 min and 60 min. Media without 1-octen-3-ol was used as a control. At each time point, the thalli were wiped dry and frozen in liquid nitrogen and stored at − 70 °C for future experiments.

### Detection of rot in thalli

The rehydrated and activated thalli were cut into 0.5 × 0.5 cm segments and then dipped into sterilized seawater in a 6-well plate at a density of 7 mg/mL (*n* = 10). 1-Octen-3-ol was added to the wells at concentrations of 0, 10, 50 and 100 μM, and maintained at 20 °C, using 40 μM photons m^− 2^ s^− 1^ under a 12:12 h (L/D) photoperiod for 7 days. Every day, the thalli were collected, cleaned with sterilized seawater, and then the percentage of rot on the thalli was recorded (*n* = 10). Rot is defined as bleaching on the thallus surface.

### Investigation of 1-octen-3-ol effects on associated bacteria

In order to quantify the impact of the algal response to 1-octen-3-ol on natural epiphytic bacterial flora, visually healthy algae were treated as described above. At each time point, the thalli were collected and washed with sterilized seawater and then homogenized using 2 mL of sterilized seawater in sterilized mortars on ice. Homogenates were diluted 100 times with sterilized seawater. After centrifugation, the supernatants were plated on nutrient agar plates (medium 2216 containing 15 g/L agar) and incubated at 20 °C. Bacteria that were associated with the algal biomass were quantified by counting the number of bacteria colonies, and then expressed as colony-forming units using standard methods. The percentage of the inhibitory rate was calculated in comparison to the control group (*n* = 10).

### Hydrogen peroxide measurement

Samples were exposed to different concentrations of 1-octen-3-ol over different periods of time, and the associated media were collected. The amount of H_2_O_2_ was measured by the dimerization of *p*-hydroxyphenyl acetic acid in the presence of horseradish peroxidase. An 8-μL aliquot of stock solution (6.13 μM *p*-hydroxyphenyl acetic acid, 276.9 U/L horseradish peroxidase, and 8.6 mM Tris-HCl, pH 8.8) was added to 200 μL of the media and incubated for 15 min without light. The signals were detected with excitation and emission fluorescence at wavelengths of 313 and 400 nm using, respectively, using Varioskan Flash (Thermo Scientific). The H_2_O_2_ concentration was calculated according to Miller’s method [46].

### Real-time quantitative PCR analysis

After treatment with 1-octen-3-ol, total RNA of samples was isolated using an AxyPre Multisource Total RNA Miniprep Kit (Axygen Bioscientific, Inc. Union City, CA, USA) and reverse-transcribed into cDNA using a Takara PrimeScript RT reagent Kit (Takara, Shiga, Japan). Real-time qRT-PCR was performed using SYBR Premix Ex Taq II (Takara) with a Mastercycle ep realplex real-time PCR system (Light Cyber 96 system, Roche Diagnostics, Switzerland). The primers for *Phlox*, *Phlox2*, *Phsod* and *Phrboh* fragments are listed in Table 4. *Ph18S* was used as internal reference gene. PCR conditions were: denature at 95 °C for 3 min, 40 cycles of denaturation at 95 °C for 15 s and annealing at 55 °C for 20 s, then a final extension at 72 °C for 10 s. Dissociation curve analysis was used to determine target specificity. Relative gene quantification was performed using the comparative 2^-ΔΔCt^ method and normalized to *Ph18S*.

**Table 4:** Primer sequences of the target genes for qRT-PCR

Genes	Primer sequence 5 → 3′	Length of fragments
*Phsod*-QF	GCTGATGGAGGGCATTGTC	158 bp
*Phsod*-QR	CGGTGTAGTTCTTGGCAATGA TGCCGCTCAAGACGACCTA	
*Phboh*-QF		90 bp
*Phboh*-QR	CACCCACCACAGACCCAGA	
*Phlox*-QF	TGCCCCACTTCGCCGACACC	130 bp
*Phlox*-QR	GCCGCCGAGAAGACGTCCATCC	
*Phlox2*-QF	TCCTTCGTGCTCTTGTTGGTT	101 bp
*Phlox2*-QR	GCTGCTGTTGTTGGGTTCCT	
*Ph18S*-QF	AGTTAGGGGATCGAAGACGA	153 bp
*Ph18S*-QR	CAGCCTTGCGACCATACTC	

### Antioxidant activity assays

Thalli treated with 1-octen-3-ol were ground into a paste using ice-cold PBS buffer. The homogenates were centrifuged at 13,000×*g* for 10 min at 4 °C. Superoxide dismutase (SOD), and glutathione peroxidase (GSH-Px) activities were measured using Assay Kits (Beyotime Biotechnology) according to the manufacturer’s instructions. One unit of SOD activity was defined as the amount of enzyme needed to exhibit 50% dismutation of superoxide radicals. One unit of GSH-Px enzyme activity was defined as the amount of enzyme that caused the oxidation of 1 μmol NADPH to NADP per min at 25 °C.

### Analysis of phospholipase activity

Phospholipase A2 (PLA_2_) activity was measured according to Chandra et al. (1996) [47]. Briefly, the rehydrated and activated thalli were cut into pieces, and 4.5 mg of thalli were placed into 96-well plates with 200 μL sterilized seawater. 0.1 μL of a stock of N-((6-(2,4-dinitrophenyl) amino)hexanoyl)-2-(4,4-difluoro-5,7-dimethyl-4-bora-3a,4adiaza-s-indacene-3-pentanoyl)-1-hexadecanoyl-sn-glycero-3-phosphoethanolamine, triethylammonium salt (PED6, Molecular Probes, Leiden, Netherlands, 1 mg/mL) in dimethylsulfoxide was added. After the addition of 50 μM 1-octen-3-ol, the fluorescence signal was monitored at 30-s steps for 1 h. The reaction temperature was kept under 18 °C with excitation and emission wavelengths of 488 and 516 nm, respectively.

### Lipid analysis by LC-MS

Samples were extracted according to the method of a Bligh and Dyer [48]. Mass spectrometry analysis was performed using a Waters Xevo G2-S Q-TOF mass spectrometer operating system in both positive and negative electrospray ionization (ESI) modes, coupled with an acquity UPLC BEH C18 analytical column (100 mm × 2.1 mm × 1.7 μm) at 40 °C, and the sample chamber temperature was 4 °C. In positive mode, the elution gradient consisted of 1 μM sodium formate in ultrapure water (solvent A) and 1 μM sodium formate in acetonitrile (solvent B) at a flow rate of 300 μL/min. In negative mode, the elution gradient consisted of 15 mM ammonium acetate in ultrapure water (solvent A) and 15 mM ammonium acetate in acetonitrile (solvent B) at a flow rate of 300 μL/min, with 0.1% formic acid in the solution. The mobile phase B was changed from 2 to 50% in 3 min, was increased to 98% in 12 min and held for 2 min, then returned to the initial 2% in 1 min and equilibrated for 2 min.

The ionization conditions were performed as follows: cone gas pressure (N_2_) flow-rate at 50 L/h; desolvation gas pressure (N_2_) flow-rate at 800 L/h; spray voltage was set as 2500 V and 3000 V in negative and positive mode, respectively; source temperature at 100 °C; desolvation temperature at 350 °C. The mass spectrometer scanned from *m/z* 50–1000. MS/MS analysis was carried out using a collision energy of 30 eV. The LC-MS data were analyzed by MassLynx (v4.2) software (Waters, USA). Identification analysis of each lipid was achieved according to previous studies with minor modification [30].

The final table containing both positive ion mode and negative ion mode was exported into SIMCA-P software package version 13.0 (Umetrics, Umea, Sweden) for multivariate data analysis [49]. A pareto scaling was applied to the variables prior to unsupervised multivariate analysis principle component analysis (PCA) and supervised partial least squares-discriminant analysis (PLS-DA). PCA was performed initially to detect intrinsic trends within the control and 1-octen-3-ol groups, separately, as an objective method. Sequentially PLS-DA was applied to achieve the maximum separation among groups with a 200 times permutation test conducted to guarantee the quality of the multivariate models and to measure over-fitting of the models. The lipid metabolites with the largest variable importance in the projection values (values > 1) from the PLS-DA analysis were considered to be influential for the separation of samples. Moreover, multi experiment viewer was employed via a Kruskal-Wallis test in order to determine whether the differential metabolites acquired from the PLS-DA models were statistically significant (*P* < 0.05) among groups or at the univariate analysis level [49].

### Analysis of free fatty acids by GC-MS

Free fatty acids (FFAs) were extracted from the 1-octen-3-ol-treated *P. haitanensis* tissues according to Küpper et al. [27]. The obtained samples were dried by nitrogen gas. Fatty acids were derivatized by adding 100 μL acetonitrile, 20 μL N,N-diisopropylethylamine and 10 μL PFBBr at 35 °C for 30 min. The sample was then dried with N_2_, dissolved in 500 μL hexane and analyzed by QP2010 GC-MS (Shimadzu, Japan).

The GC/MS analysis was performed using an SPB-50 fused silica capillary column (30 m × 0.25 mm × 0.25 μm, Supelo, Bellefonte, PA, USA). The injection port temperature was 250 °C, helium gas flow was 0.81 mL/min, the pre-column pressure was constant at 73.0 kPa, the mass spectrometer ion source temperature was 200 °C, the interface temperature was 250 °C, the electron energy was 70 eV, and ionization pattern was for electron ionization. The mass spectrometer scanned from 50 to 600 m/z. The temperature program was 150 °C for 3.5 min, raised to 200 °C for 5 min at a rate of 20 °C/min, and finally raised from 250 °C to 280 °C for 30 min at a rate of 5 °C/min. The injection volume was 1 μL and the split ratio was 50:1. The compounds were ionized by negative ion chemical ionization using methane as the reagent gas. The mass spectrometer scanned from *m/z* 50 to 600. Fatty acids were quantified as pentafluorobenzyl esters from standard curves obtained by measuring the peak areas of authentic standards.

### Analysis of oxylipins by HPLC-QE-MS

Samples (0.5 g fresh weight) were extracted with 1 mL of ethyl acetate under 4 °C for 1 h. The supernatant of organic phase was obtained by centrifugation (12,000 rpm for 10 min, 4 °C). After performing the extraction three times, deionized ice water was added and the mixture was shaken vigorously to remove water-soluble impurities. The mixture was incubated on ice for 5 min and then centrifuged at 4 °C for 10 min to obtain the organic solvent, which was evaporated by nitrogen gas. The residue was dissolved in 500 μL methanol.

LC-MS/MS analysis was performed using a Finnigan Surveror and TSQ Quantum Access system (Thermo Fisher Scientific, San Jose, CA, USA) equipped with ESI and interfaced with a triple quadrupole mass spectrometer. A hypersil gold C18 column (100 mm × 2.1 mm × 1.7 μm) was used at 30 °C. The elution gradient consisted of 0.2% acetic acid in ultrapure water (solvent A) and acetonitrile (solvent B) at a flow rate of 200 μL/min. Elution was performed by shifting from 30 to 100% acetonitrile in 40 min, then to 30% acetonitrile in 1 min, followed by 9 min of re-equilibration. The analysis parameters of ionization conditions were adjusted as follows: the data dependent mode switched between full scan MS and MS/MS acquisition in the negative ion mode. Sheath gas pressure (N_2_) flow-rate at 30 L/min; aux gas pressure (N_2_) flow-rate at five Abs; spray voltage at 2500 V; vaporizer temperature at 300 °C; capillary temperature at 320 °C. Argon was introduced into the trap with an estimated pressure of 6 × 10^− 6^ mbar to improve trapping efficiency and to act as the collision gas for full scan mode. The collision gas pressure was 1.5 mTorr. Results are presented as peak area.

### Analysis of volatile compounds by GC-MS

Fresh samples (0.3 g) were ground in 2 mL potassium phosphate buffer (50 m mol/L Tris and 20 m mol/L NaCl, pH = 8.0) on ice, and then extracted using solid-phase microextraction fiber (Supelco, Bellefonte, PA, USA) coated with an absorbent phase made of polydimethysiloxane/carboxen/divinylbenzene under headspace mode at 40 °C for 50 min with stirring. Vanillin was used as a reference standard. After extraction, the extraction device was inserted into the injection port and maintained for 5 min at 250 °C. Analysis was performed using a Shimadzu QP2010 GC-MS equipped with a vocol column (60 m × 0.32 mm × 0.18 μm, Supelco, Bellefonte, PA, USA). Helium was used as the carrier gas at a constant flow rate of 0.81 mL/min. The program was set as 35 °C for 3 min, then raised to 40 °C at 3 °C/min and held for 1 min, before finally being raised to 210 °C at 5 °C/min and held for 25 min. The electron ionization system at an ionization energy of 70 eV was used, and the mass spectra scan ranged from *m/z* 45 to 1000. Identification was conducted based on comparison of molecular weights and mass spectra fragmentation patterns with those recorded in the Nist 147 and Wiley 7 Spectrometry Library data (GC/MS), utilizing previous analyses of pure references that are commercially available.

### Analysis of phytohormones by LC-MS

The extraction of phytohormones and identification were conducted as described by Wang et al. with minor modifications [39]. Samples (3 g) were lyophilized and ground under liquid nitrogen. The powder was extracted twice with acetonitrile:water:formic acid (80:19:1, *V*/V/V) containing an antioxidant (0.5% butylated hydroxytoluene) by ultrasonication for 10 min and stored at − 20 °C without light for 16 h. The supernatant phase obtained by centrifugation was dried by vacuum evaporation at 20 °C and then dissolved in 500 μL of MeOH/H_2_O/CH_3_COOH (90:10:0.05, V/V/V), followed by filtration using a 0.22-μm membrane for LC-MS analysis (Thermo Fisher Scientific, Rockvile, MD, USA).

Analysis was performed using a Finnigan Surveyor and TSQ Quantum Access equipped with an ESI mass spectrometer. A hypersil gold C18 column (100 mm × 2.1 mm × 1.7 μm) was used at 30 °C with a gradient elution program of 10 mM ammonium acetate-methanol at a flow rate of 300 μL/min. Elution was performed with 15–95% methanol for 10 min, maintained for 1 min. The ionization conditions were the same as those used for oxylipin detection. The LC-MS was operated in positive and negative modes.

The commercial standards for all plant hormones were purchased from Sigma-Aldrich (St. Louis, MO, USA) to establish the quantitative standard curves. The hormones in the samples were identified by comparing the retention time and MS information with those of the standards.

### Statistical analysis

All experiments were performed in triplicate. Statistical analyses were performed using SPSS software, version 16.0 (SPSS Inc., Chicago, IL, USA). The results are presented as mean values ± standard deviation (SD) and the statistical significance was analyzed by one-way ANOVA. *P* values less than 0.05 were considered to be statistically significant.

## Additional files

Additional file 1: **Figure S1.** The base peak ionization chromatogram of a *Pyropia haitanensis* sample. (PDF 369 kb)

Additional file 2: **Figure S2.** The score plot of principal component analysis (PCA) of lipid profiles in *Pyropia haitanensis* extracts cultured under control and 1-octen-3-ol treatment. A, Positive; B, Negative. (PDF 326 kb)

Additional file 3: Differential lipid identification. (PDF 17 kb)

Additional file 4: **Table S1.** Lipidomics response of *Pyropia haitanensis* to 1-octen-3-ol treatment. (PDF 29 kb)

Additional file 5: **Figure S3.** MS/MS identification of oxylipins. (PPTX 338 kb)

Additional file 6: **Figure S4.** MS/MS identification of methyl jasmonic acid. (PDF 406 kb)

## Abbreviations

DGDG: Digalactosyldiacylglycerol
FFA: Free fatty acid
GA3: Gibberellin A3
GSH-Px: Glutathione peroxidase
HEPE: Hydroxy eicosapentaenoic acid
HETE: Hydroxy eicosatetraenoic acid
HOTE: Hydroxy octadecatrienoic acid
HpEPE: Hydroperoxy eicosapentaenoic acid
HpETE: Hydroperoxy eicosatetraenoic acid
HPL: Hydroperoxide lyase
HpODE: Hydroperoxy octadecadienoic acid
HpOTE: Hydroperoxy octadecatrienoic acid
IAA: Indole-3-acetic acid
LOX: Lipoxygenase
MeJA: Methyl jasmonic acid
PA: Phosphatidic acid
PC: Phosphatidylcholine
PCA: Principle component analysis
PE: Phosphatidylethanolamine
PG: Phosphatidylglycerol
PI: Phosphatidylinositol
PLA_2_: Phospholipase A2
PLS-DA: Partial least squares-discriminant analysis
PUFA: Polyunsaturated fatty acid
SOD: Superoxide dismutase
SQDG: Sulfoquinovosyldiacylglycerol
