# Supplementary figures and images for: 1-Octen-3-ol, a self-stimulating oxylipin messenger, can prime and induce defense of marine alga
Source: BMC Plant Biol. 2019 Jan 22;19:37. doi: 10.1186/s12870-019-1642-0 (PMC6341616; doi:10.1186/s12870-019-1642-0)

20150107lipidomics-QC-5-

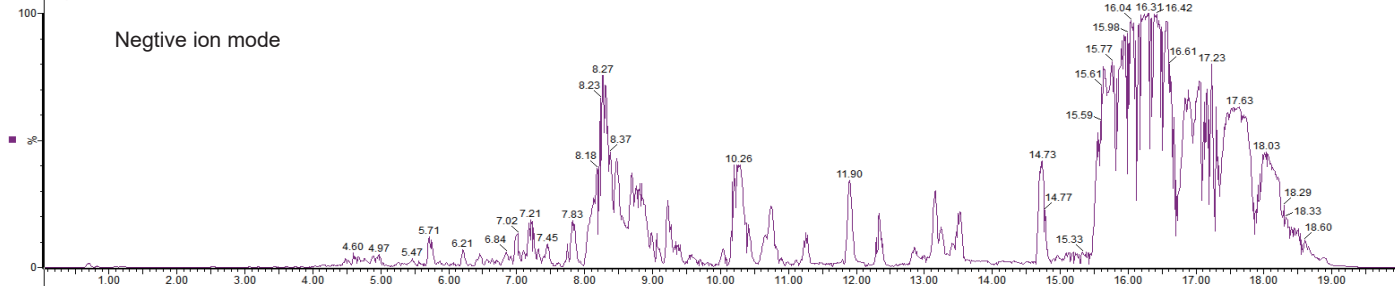

20150107lipidomics-QC-5

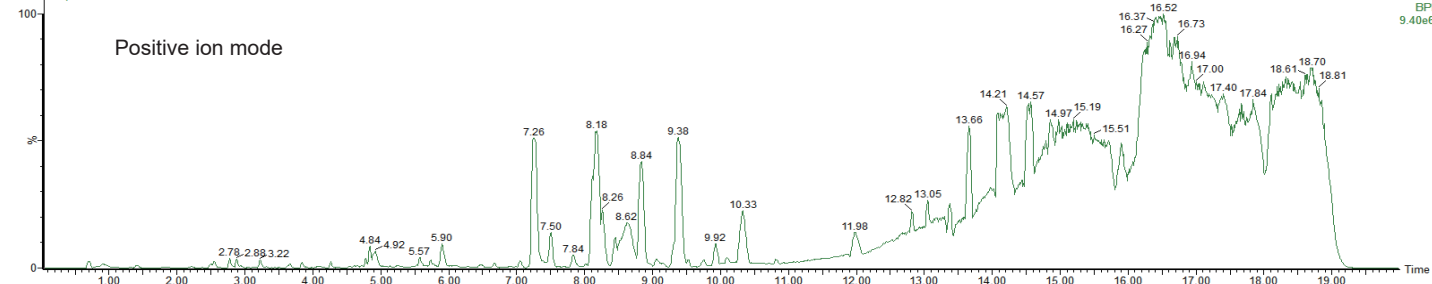

Supplement: Supplementary file 1 — Figure S1. The base peak ionization chromatogram of a Pyropia haitanensis sample. (PDF 369 kb) [file 12870_2019_1642_MOESM1_ESM.pdf]

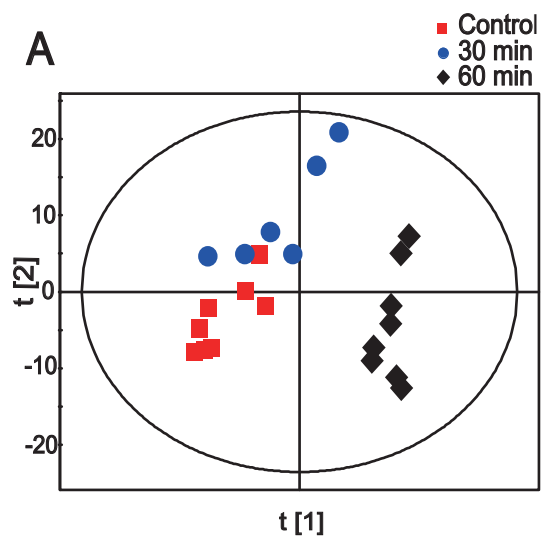

Supplement: Supplementary file 2 — Figure S2. The score plot of principal component analysis (PCA) of lipid profiles in Pyropia haitanensis extracts cultured under control and 1-octen-3-ol treatment. A, Positive; B, Negative. (PDF 326 kb) [file 12870_2019_1642_MOESM2_ESM.pdf]

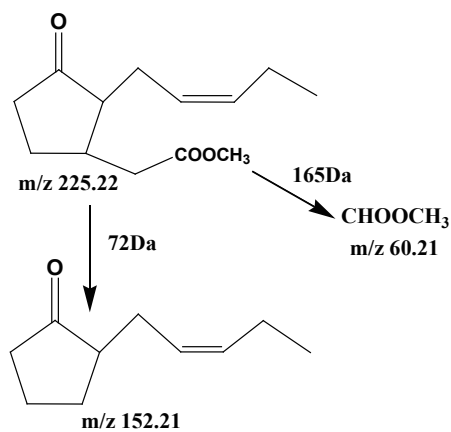

Methyl jasmonic acid

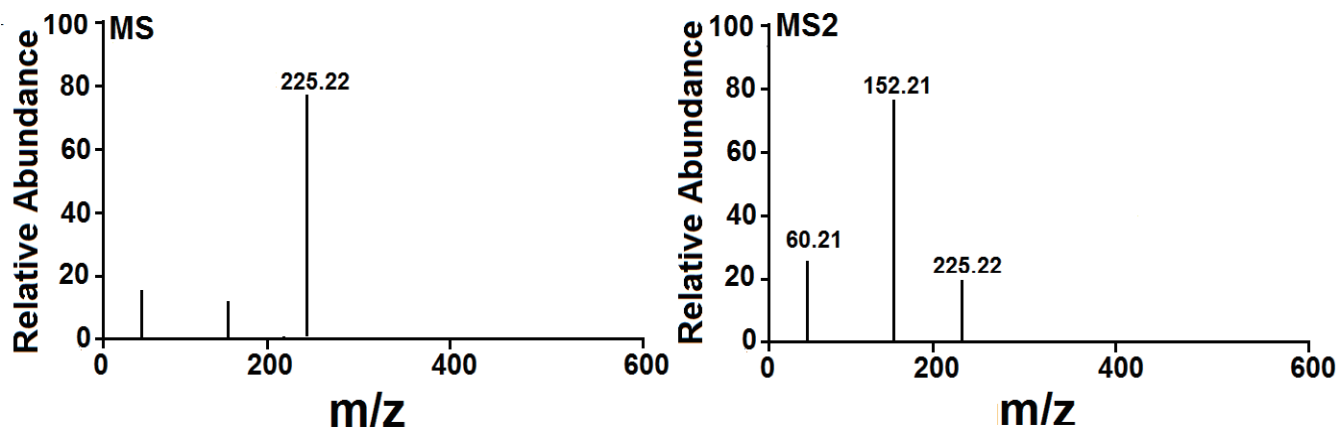

Supplement: Supplementary file 6 — Figure S4. MS/MS identification of methyl jasmonic acid. (PDF 406 kb) [file 12870_2019_1642_MOESM6_ESM.pdf]
