# Supplementary material for: 1-Octen-3-ol, a self-stimulating oxylipin messenger, can prime and induce defense of marine alga
Source: BMC Plant Biol. 2019 Jan 22;19:37. doi: 10.1186/s12870-019-1642-0 (PMC6341616; doi:10.1186/s12870-019-1642-0)
Supplement: Supplementary file 3 — Differential lipid identification. (PDF 17 kb) [file 12870_2019_1642_MOESM3_ESM.pdf]

Identification of complex lipids in lipidomic analyses

DGDG efficiently produced $[M+Na]^+$ ions in the positive mode [1], whereas SQDG, PE and PG yielded deprotonated $[M-H]^-$ ions in the negative mode [2]. High levels of PC was observed; these formed $[M+H]^+$ and $[M-H]^-$ in both positive and negative modes [3]. Fragment ions containing a digalactosyl head group ($[C_{15}H_{26}O_{11}+Na]^+$, m/z 405.1333, 8.4ppm) was used as characteristic fragment ion of DGDG [1]. Fragment ion with the sulfoquinovosyl head group ($[C_6H_9O_7S]^-$, m/z 225.0084, 6.7ppm) was used as characteristic fragment ion of SQDG [3]. Fragment ion generated by eliminating a molecule of H_2O ($[C_6H_{12}O_7P]^-$ at m/z 227.0341, 8.8ppm) from glycerophosphate glycerol and fragment ion of glycerol phosphate($[C_3H_8O_6P]^-$, m/z 171.0041, 9.9ppm) were used for PG identification [1]. The neutral loss (141Da) of head group of phosphoethanolamine was used for PE identification [3]. Fragment ions of $[C_5H_{15}O_4NP]^+$ at m/z 184.0748(4.9ppm) and $[C_4H_{11}O_4NP]^-$ at m/z 168.0442 (9.5ppm) were used for PC identification in positive and negative ion mode, respectively [1, 4]. Besides, the location of two acyl chains of lipids could also be determined based on the ratio of sn-1 to sn-2 carboxylate fragment ions abundances. For DGDG, the abundance of sn-1 carboxylate fragmentations are stronger than that of the sn-2 carboxylate ions; while for SQDG, PC, PE and PG, the abundance of sn-1 carboxylate fragment ions was weaker than that of the sn-2 carboxylate ions [1, 2, 5]. Based ononline lipid databases and fragmentation pathways, a total of 25 differential lipids were identified (Table S1).

References

[1] Xu JL, Chen DL, Yan XJ, Chen JJ, Zhou CX. Global characterization of the photosynthetic glycerolipids from a marine diatom *Stephanodiscus sp.* by ultra performance liquid chromatography coupled with electrospray ionization-quadrupole-time of flight mass spectrometry. Anal Chim Acta.

2010, 663(1): 60-8.

[2] Yan XJ, Li HY, Xu JL, Zhou CX. Analysis of phospholipids in microalga *Nitzschia closterium* by UPLC-Q-TOF-MS. J Oceanol Limnol. 2010, 28(1): 106-12.

[3] Pulfer M, Murphy RC. Electrospray mass spectrometry of phospholipids. Mass Spectrom Rev. 2003, 22(5): 332-64.

[4] Yan XJ, Xu JL, Chen JJ, Chen DY, Xu SL, Luo QJ, Wang YJ. Lipidomics focusing on serum polar lipids reveals species dependent stress resistance of fish under tropical storm. Metabolomics. 2012, 8(2): 299-309.

[5] Guella G, Frassanito R, Mancini I. A new solution for an old problem: the regiochemical distribution of the acyl chains in galactolipids can be established by electrospray ionization tandem mass spectrometry. Rapid Commun Mass Spectrom. 2003, 17(17): 1982–94.
