# Supplementary material for: 1-Octen-3-ol, a self-stimulating oxylipin messenger, can prime and induce defense of marine alga
Source: BMC Plant Biol. 2019 Jan 22;19:37. doi: 10.1186/s12870-019-1642-0 (PMC6341616; doi:10.1186/s12870-019-1642-0)
Supplement: Supplementary file 4 — Table S1. Lipidomics response of Pyropia haitanensis to 1-octen-3-ol treatment. (PDF 29 kb) [file 12870_2019_1642_MOESM4_ESM.pdf]

Table S1 Lipidomics response of *Pyropia haitanensis* to 1-octen-3-ol treatment.

Compound	VIP (30 min)	*P* value (30 min/Control)	Fold change (30 min/Control)	VIP (60 min)	*P* value (60 min/Control)	Fold change (60 min/Control)
**PC (20:5/20:5)**	19.1	1.43E-03	-2.61	15.72	2.08E-05	-4.61
**PC (16:0/18:1)**	5.06	8.53E-02	-1.78	4.95	4.55E-03	-2.69
**PC (18:1/20:6)**	2.92	4.13E-02	-0.78	7.12	1.65E-04	-3.77
**PC (16:0/20:5)**	13.67	2.40E-03	-1.5	13.34	2.39E-06	-2.7
**PC (20:2/20:6)**	2.64	7.74E-03	-0.23	7.4	2.67E-03	-1.33
**Lyso PC (18:1)**	11.08	3.33E-02	0.52	11.24	3.94E-02	1.35
**Lyso PC (18:2)**	2.02	4.55E-02	-0.32	7.32	3.97E-03	0.5
**Lyso PC (20:3)**	14.42	1.28E-02	-1.66	0.95	2.86E-02	0.08
**Lyso PC (22:5)**	0.53	4.34E-02	-0.33	2.38	4.51E-02	0.52
**PG (16:0/20:5)**	8.71	3.92E-02	1.29	16.35	2.05E-03	2.31
**Lyso PG (20:4)**	1.03	3.94E-05	-0.42	1.64	1.83E-05	-2.12
**Lyso PG (20:5)**	1.93	4.63E-02	-0.36	1.12	2.85E-01	0.12
**Lyso PG (20:6)**	4.75	1.69E-02	-1.28	5.74	9.95E-03	-2.23
**Lyso PG (14:0)**	2.47	1.91E-02	-0.46	3.18	2.02E-03	-0.55
**Lyso PG (16:0)**	1.82	7.78E-04	-0.85	1.83	2.70E-04	-0.53
**Lyso PE (16:1)**	1.34	4.03E-05	-1.07	1.36	1.69E-05	-0.77
**Lyso PE (18:0)**	1.95	2.63E-03	-1.6	1.83	4.96E-03	-1
**Lyso PE (18:1)**	1.22	1.27E-02	-0.31	0.71	1.41E-01	0.18
**Lyso PE (22:4)**	4.45	2.70E-02	2.13	4.45	2.99E-02	2.29
**PA (20:5/20:4)**	1.04	4.43E-02	0.55	2.21	7.22E-03	1.33
**Lyso PA (20:4)**	1.11	3.36E-02	0.65	2.29	7.15E-03	1.14

**PI (16:0/18:1)**	1.67	1.30E-02	-0.59	1.77	2.50E-03	-0.32
**Lyso PI (18:2)**	4.25	2.50E-02	1.26	5.3	9.54E-03	2.28
**DGDG (20:5/16:0)**	2.58	1.81E-02	-1.32	2.71	1.42E-05	-2.02
**Lyso SQDG (18:1)**	2.89	4.93E-02	2.31	3.7	2.26E-02	2.87

Values in this table are the fold change (1-octen-3-ol-treated/control) of selected lipids on 1-octen-3-ol-treated samples.
