# Supplementary material for: 1-Octen-3-ol, a self-stimulating oxylipin messenger, can prime and induce defense of marine alga
Source: BMC Plant Biol. 2019 Jan 22;19:37. doi: 10.1186/s12870-019-1642-0 (PMC6341616; doi:10.1186/s12870-019-1642-0)
Supplement: Supplementary file 5 — Figure S3. MS/MS identification of oxylipins. (PPTX 338 kb) [file 12870_2019_1642_MOESM5_ESM.pptx]

## Slide 1
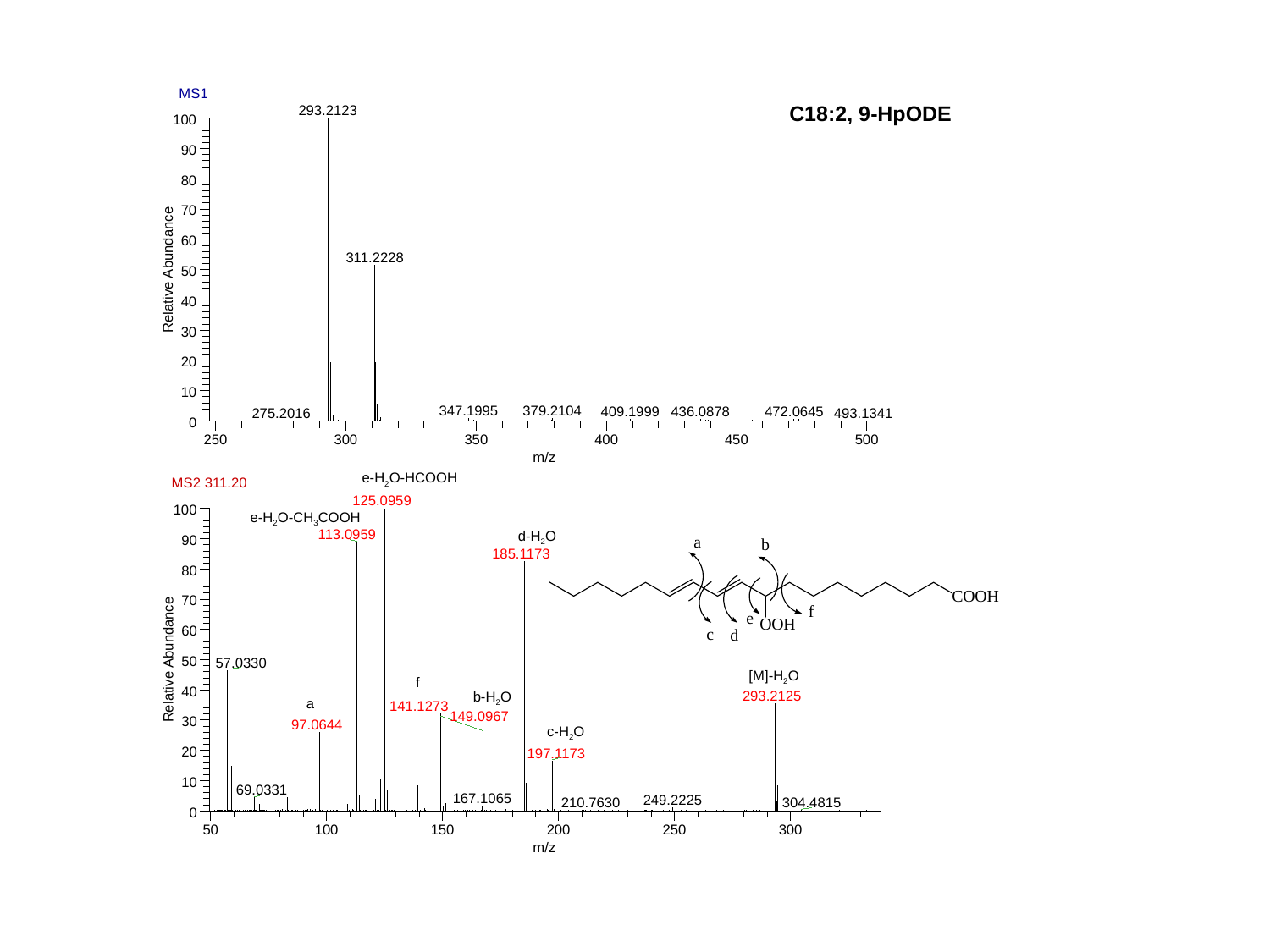

MS1
293.2123
100
90
80
70
60
311.2228
Relative Abundance
50
40
30
20
10
379.2104
347.1995
436.0878
472.0645
409.1999
275.2016
493.1341
0
250
300
350
400
450
500
m/z
C18:2, 9-HpODE
e-H2O-HCOOH
MS2 311.20
125.0959
100
e-H2O-CH3COOH
113.0959
d-H2O
90
185.1173
80
70
60
Relative Abundance
50
57.0330
f
40
293.2125
b-H2O
a
141.1273
149.0967
30
97.0644
c-H2O
20
197.1173
10
69.0331
167.1065
249.2225
210.7630
304.4815
0
50
100
150
200
250
300
m/z
[M]-H2O

## Slide 2
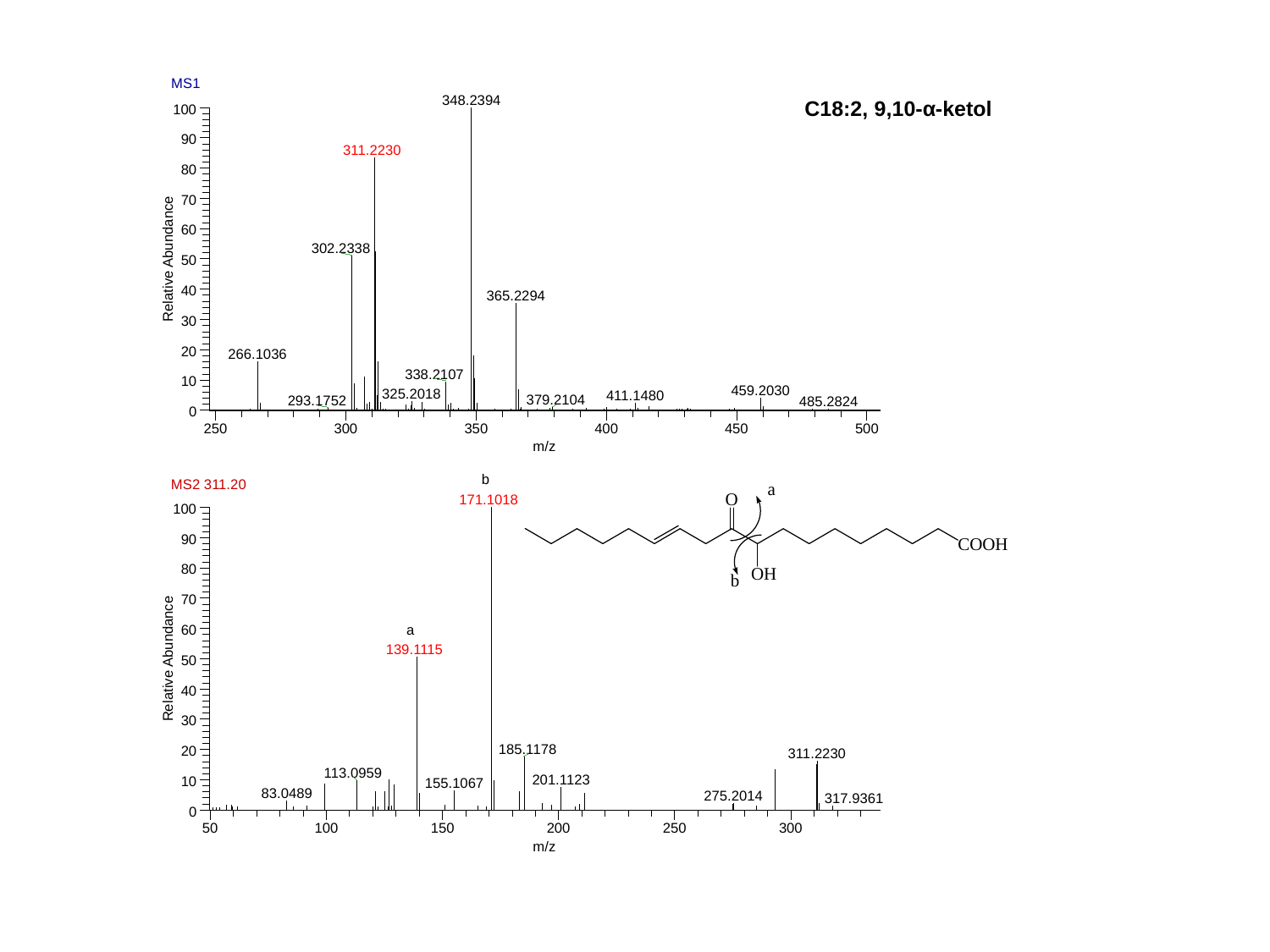

MS1
348.2394
100
90
311.2230
80
70
60
302.2338
Relative Abundance
50
40
365.2294
30
20
266.1036
338.2107
10
459.2030
325.2018
411.1480
379.2104
293.1752
485.2824
0
250
300
350
400
450
500
m/z
C18:2, 9,10-α-ketol
b
MS2 311.20
171.1018
100
90
80
70
60
a
139.1115
Relative Abundance
50
40
30
185.1178
20
311.2230
113.0959
201.1123
10
155.1067
83.0489
275.2014
317.9361
0
50
100
150
200
250
300
m/z

## Slide 3
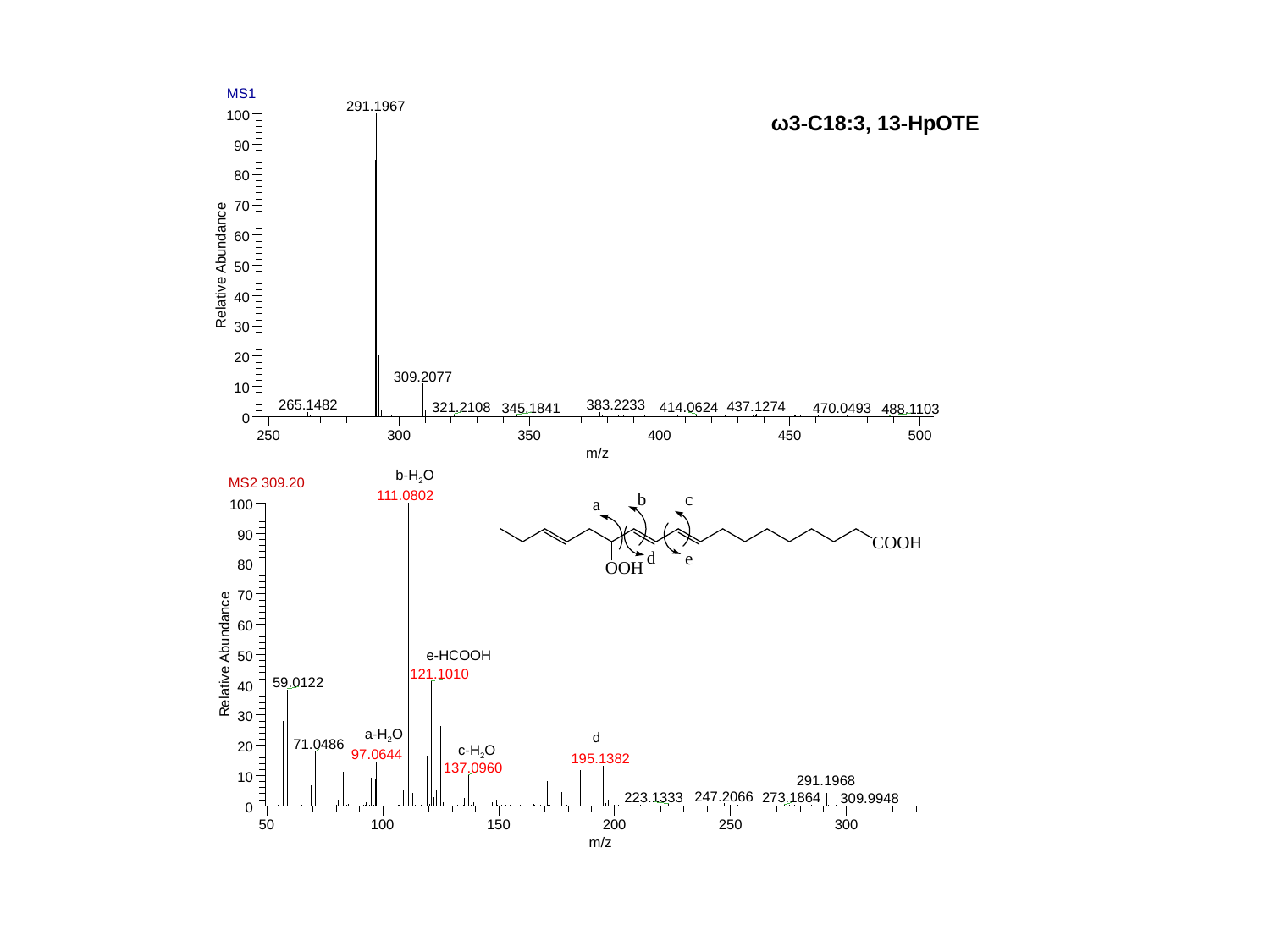

MS1
291.1967
100
90
80
70
60
Relative Abundance
50
40
30
20
309.2077
10
383.2233
265.1482
437.1274
321.2108
414.0624
345.1841
470.0493
488.1103
0
250
300
350
400
450
500
m/z
ω3-C18:3, 13-HpOTE
b-H2O
MS2 309.20
111.0802
100
90
80
70
60
Relative Abundance
e-HCOOH
50
121.1010
59.0122
40
30
a-H2O
d
71.0486
20
c-H2O
97.0644
195.1382
137.0960
10
291.1968
247.2066
223.1333
273.1864
309.9948
0
50
100
150
200
250
300
m/z

## Slide 4
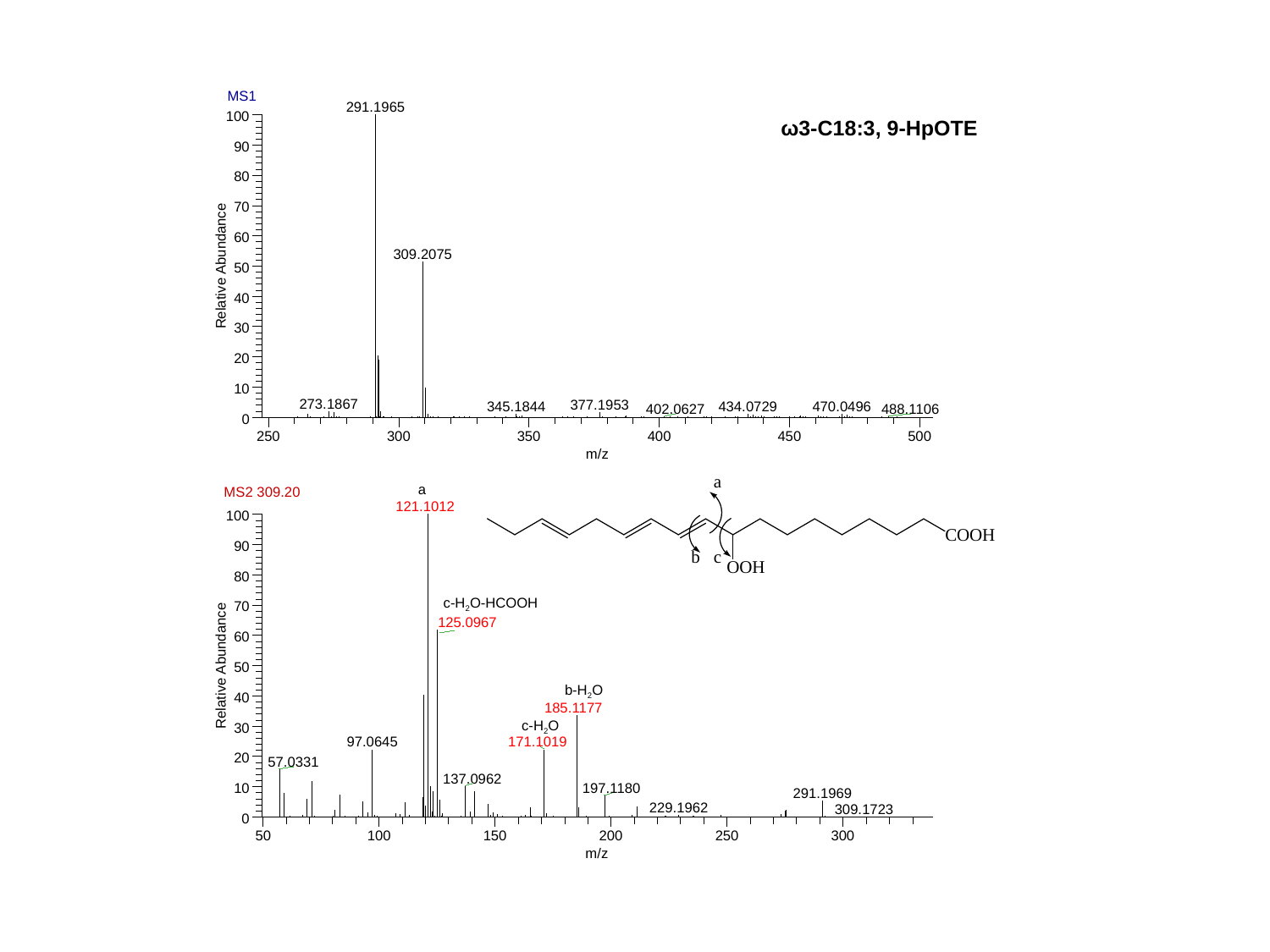

MS1
291.1965
100
90
80
70
60
309.2075
Relative Abundance
50
40
30
20
10
273.1867
377.1953
345.1844
434.0729
470.0496
402.0627
488.1106
0
250
300
350
400
450
500
m/z
ω3-C18:3, 9-HpOTE
a
MS2 309.20
121.1012
100
90
80
c-H2O-HCOOH
70
125.0967
60
Relative Abundance
50
b-H2O
40
185.1177
c-H2O
30
97.0645
171.1019
20
57.0331
137.0962
10
197.1180
291.1969
229.1962
309.1723
0
50
100
150
200
250
300
m/z

## Slide 5
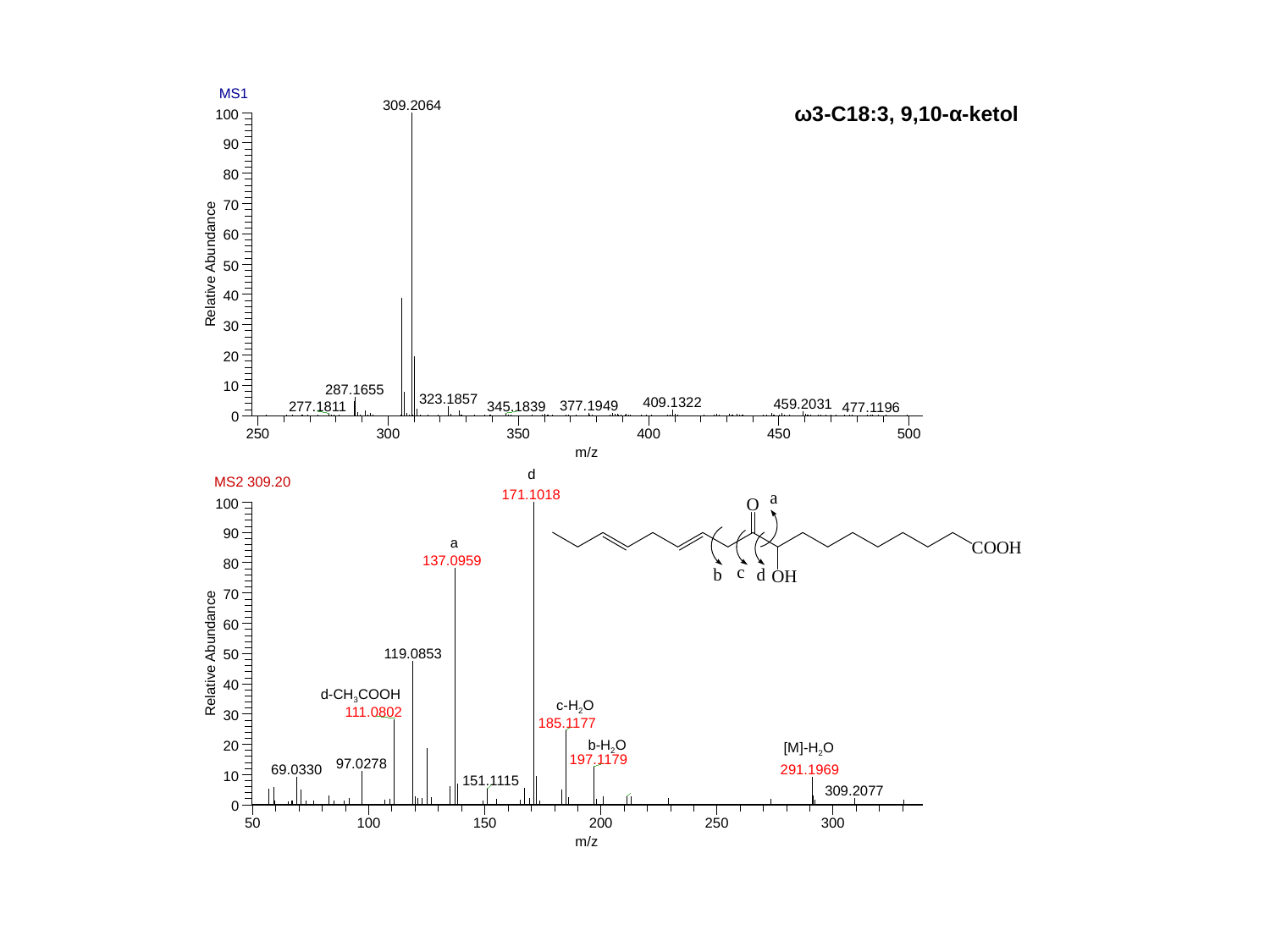

MS1
309.2064
100
90
80
70
60
Relative Abundance
50
40
30
20
10
287.1655
323.1857
409.1322
459.2031
377.1949
277.1811
345.1839
477.1196
0
250
300
350
400
450
500
m/z
ω3-C18:3, 9,10-α-ketol
d
MS2 309.20
171.1018
100
90
a
137.0959
80
70
60
Relative Abundance
119.0853
50
40
d-CH3COOH
c-H2O
111.0802
30
185.1177
b-H2O
20
[M]-H2O
197.1179
97.0278
291.1969
69.0330
10
151.1115
309.2077
0
50
100
150
200
250
300
m/z

## Slide 6
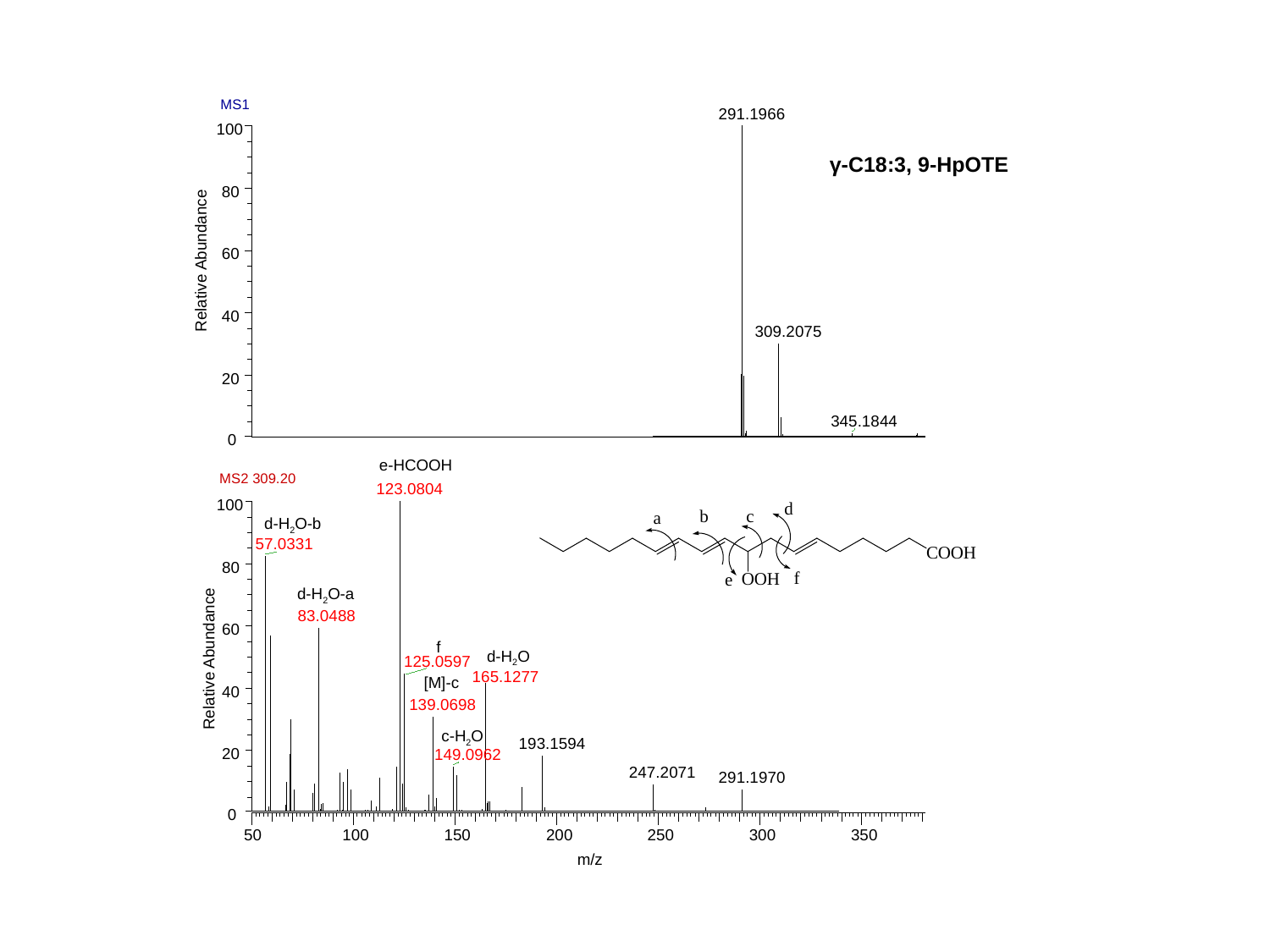

MS1
291.1966
100
80
60
Relative Abundance
40
309.2075
20
345.1844
0
γ-C18:3, 9-HpOTE
e-HCOOH
MS2 309.20
123.0804
100
d-H2O-b
57.0331
80
d-H2O-a
83.0488
60
f
d-H2O
Relative Abundance
125.0597
165.1277
[M]-c
40
139.0698
c-H2O
193.1594
20
149.0962
247.2071
291.1970
0
50
100
150
200
250
300
350
m/z

## Slide 7
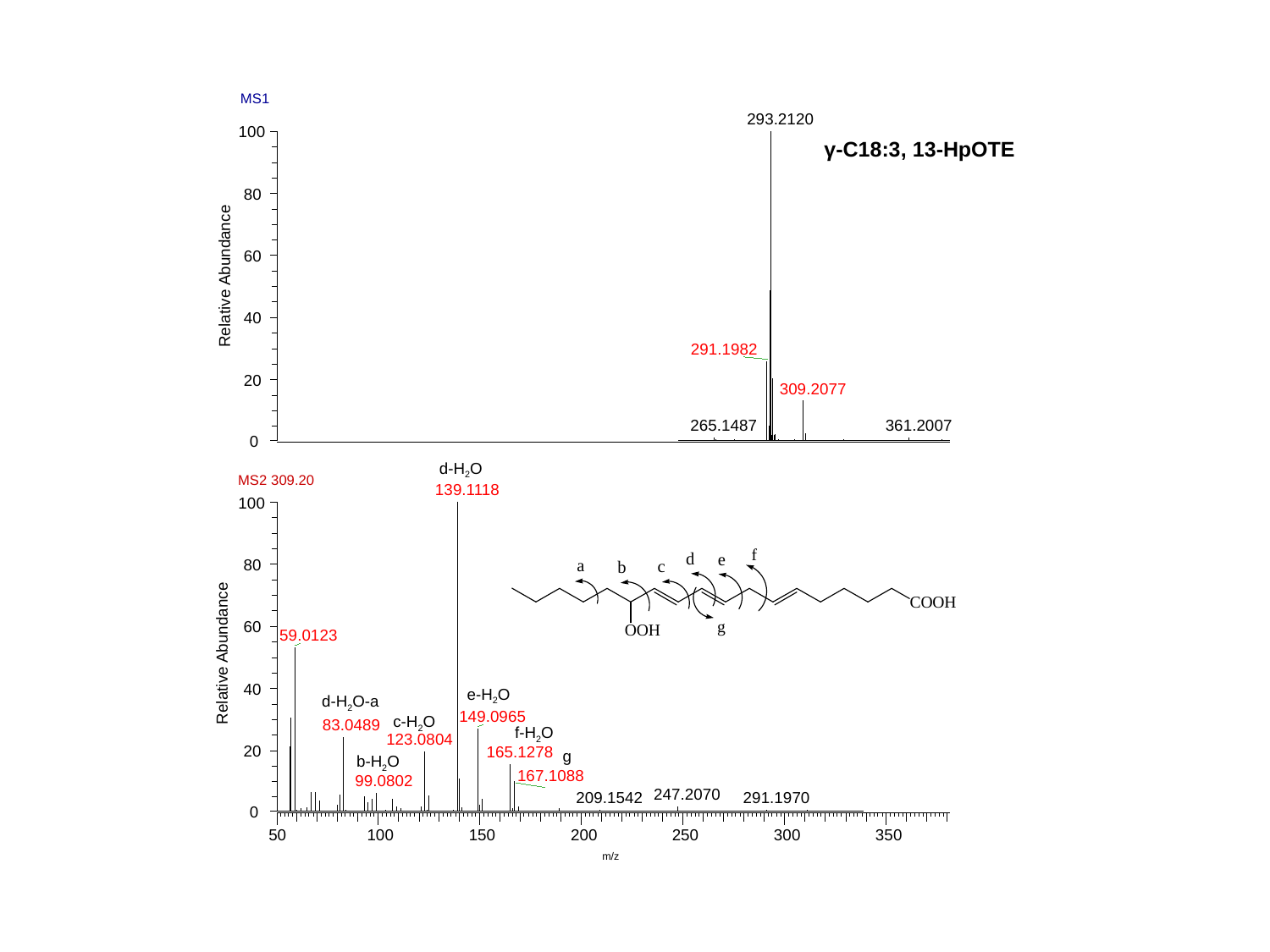

MS1
293.2120
100
80
60
Relative Abundance
40
291.1982
20
309.2077
265.1487
361.2007
0
γ-C18:3, 13-HpOTE
d-H2O
MS2 309.20
139.1118
100
80
60
59.0123
Relative Abundance
40
e-H2O
d-H2O-a
149.0965
c-H2O
83.0489
f-H2O
123.0804
20
165.1278
g
b-H2O
167.1088
99.0802
247.2070
209.1542
291.1970
0
50
100
150
200
250
300
350
m/z

## Slide 8
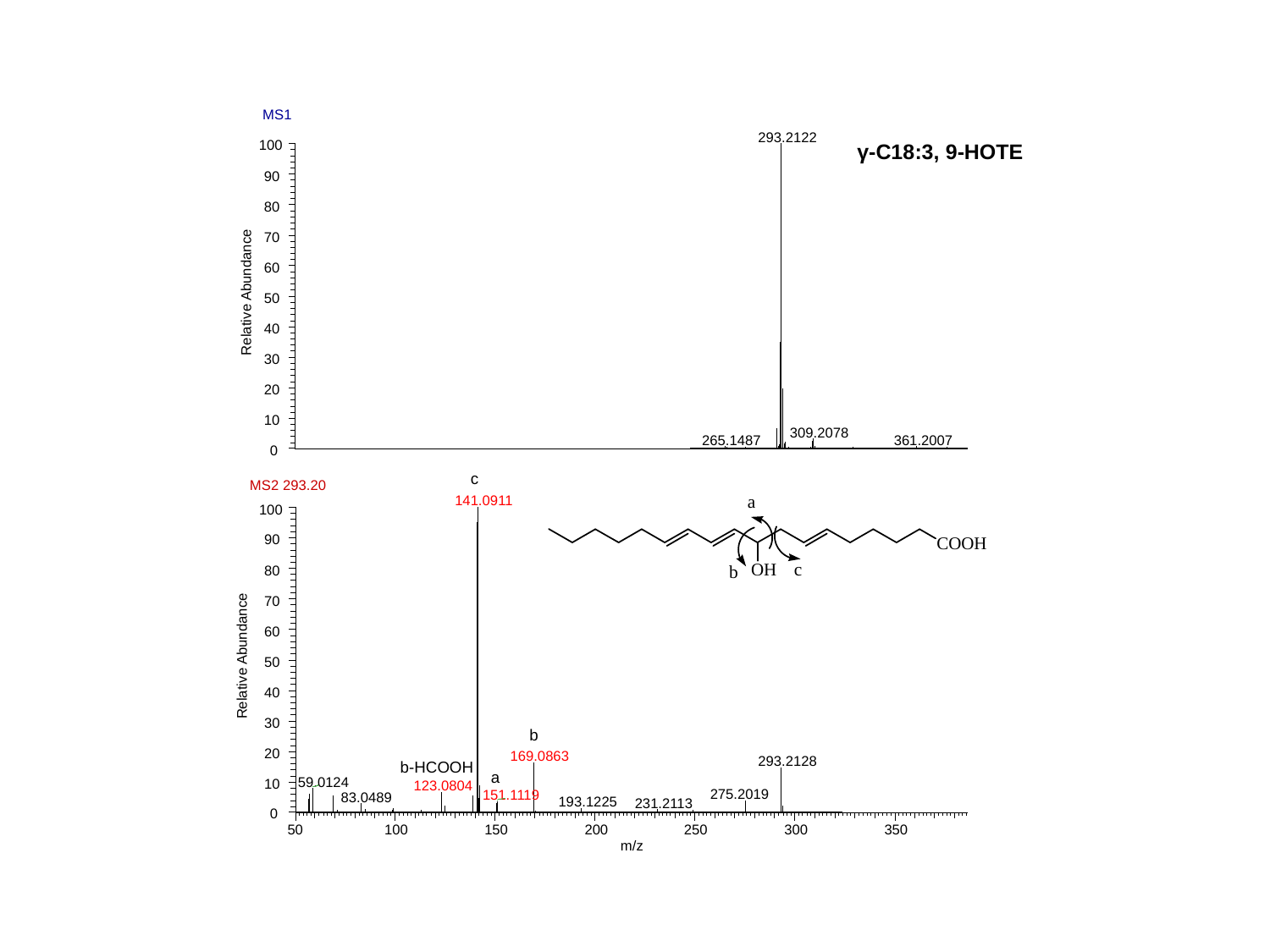

MS1
293.2122
100
90
80
70
60
Relative Abundance
50
40
30
20
10
309.2078
265.1487
361.2007
0
γ-C18:3, 9-HOTE
c
MS2 293.20
141.0911
100
90
80
70
60
Relative Abundance
50
40
30
b
20
169.0863
293.2128
b-HCOOH
a
59.0124
10
123.0804
275.2019
151.1119
83.0489
193.1225
231.2113
0
50
100
150
200
250
300
350
m/z

## Slide 9
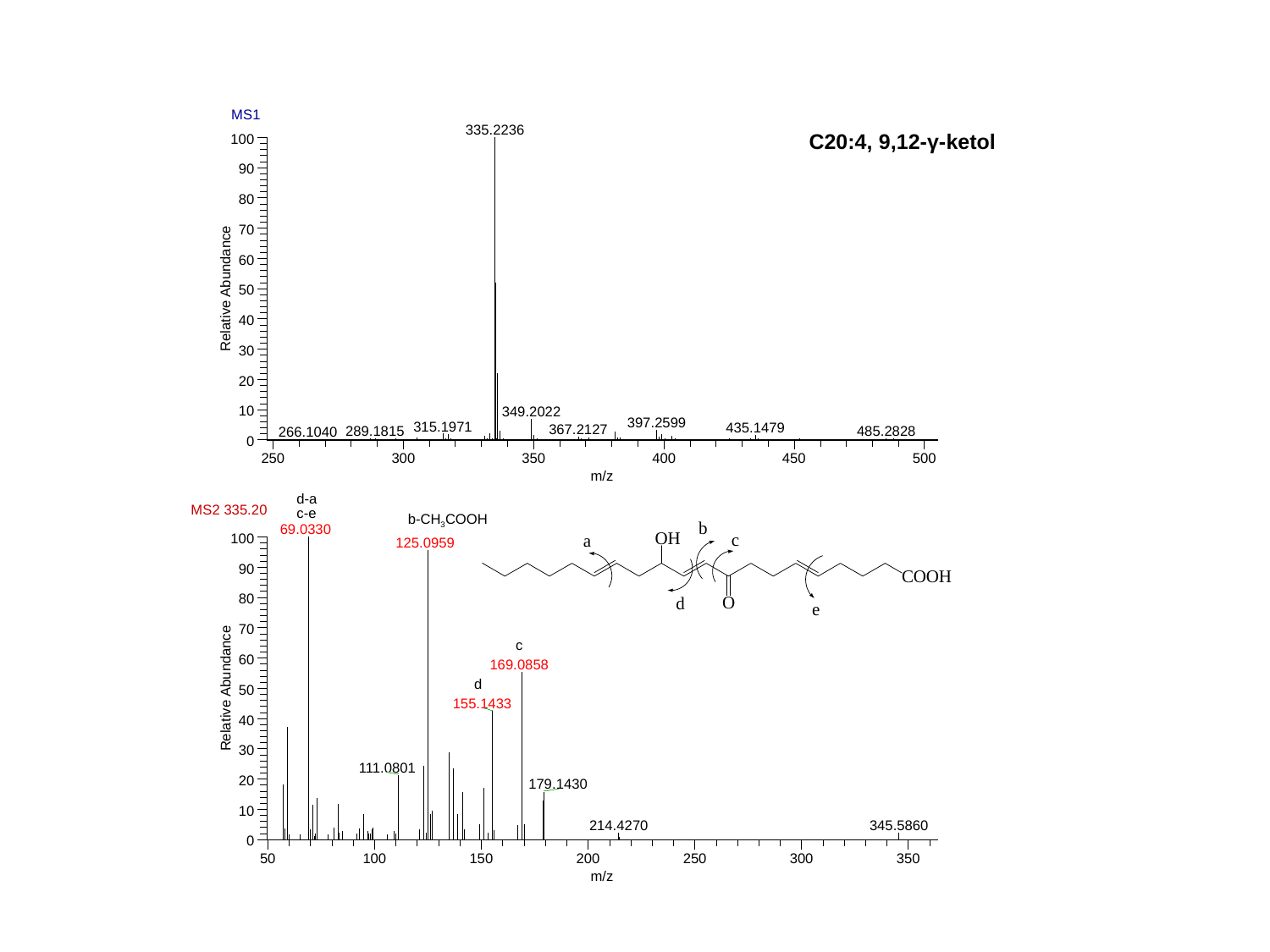

MS1
335.2236
100
90
80
70
60
Relative Abundance
50
40
30
20
10
349.2022
397.2599
315.1971
435.1479
367.2127
289.1815
485.2828
266.1040
0
250
300
350
400
450
500
m/z
C20:4, 9,12-γ-ketol
d-a
c-e
MS2 335.20
b-CH3COOH
69.0330
100
125.0959
90
80
70
c
60
169.0858
d
Relative Abundance
50
155.1433
40
30
111.0801
20
179.1430
10
345.5860
214.4270
0
50
100
150
200
250
300
350
m/z

## Slide 10
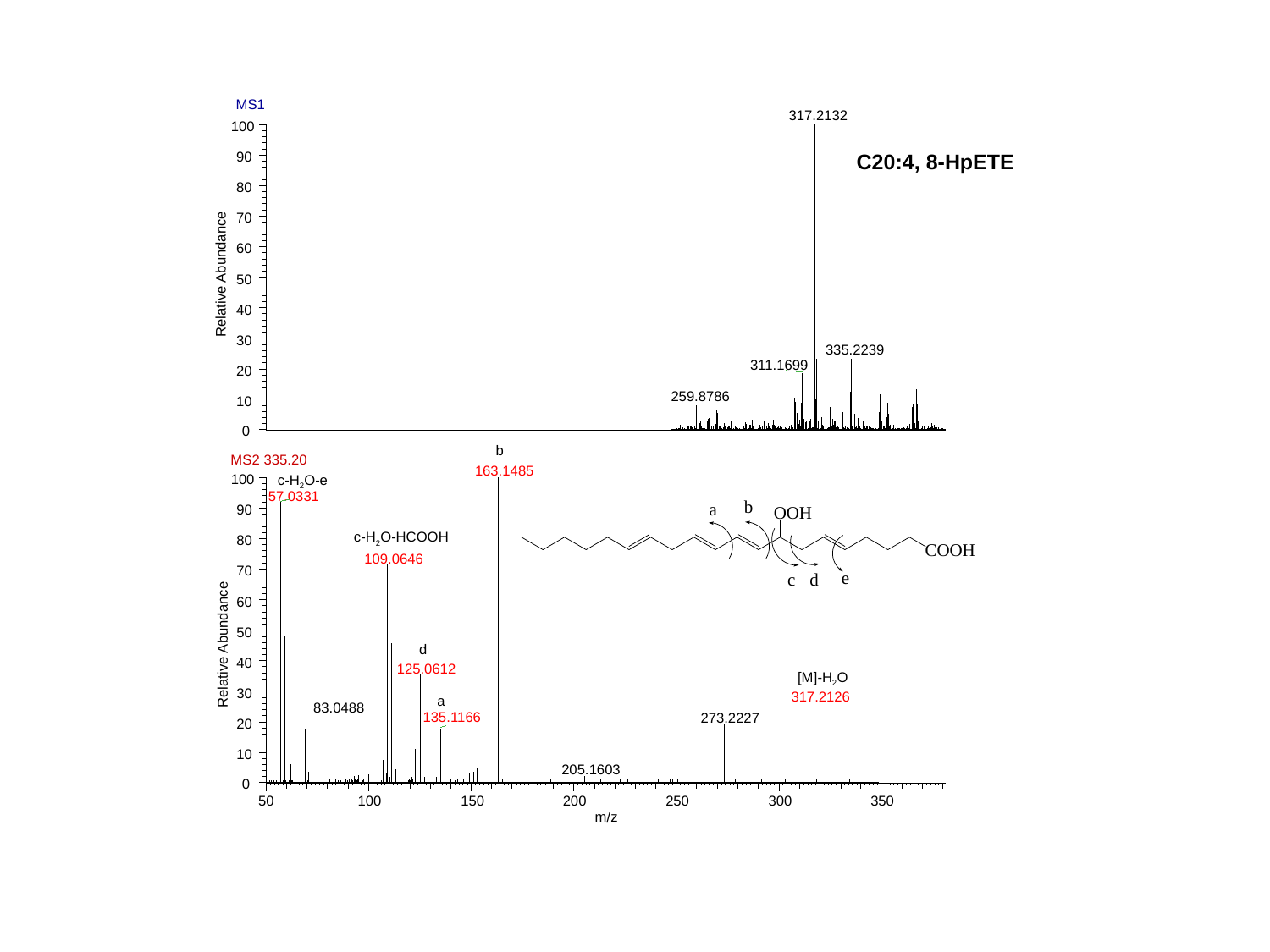

MS1
317.2132
100
90
80
70
60
Relative Abundance
50
40
30
335.2239
311.1699
20
259.8786
10
0
C20:4, 8-HpETE
b
MS2 335.20
163.1485
100
c-H2O-e
57.0331
90
c-H2O-HCOOH
80
109.0646
70
60
Relative Abundance
50
d
40
125.0612
[M]-H2O
30
317.2126
a
83.0488
135.1166
273.2227
20
10
205.1603
0
50
100
150
200
250
300
350
m/z

## Slide 11
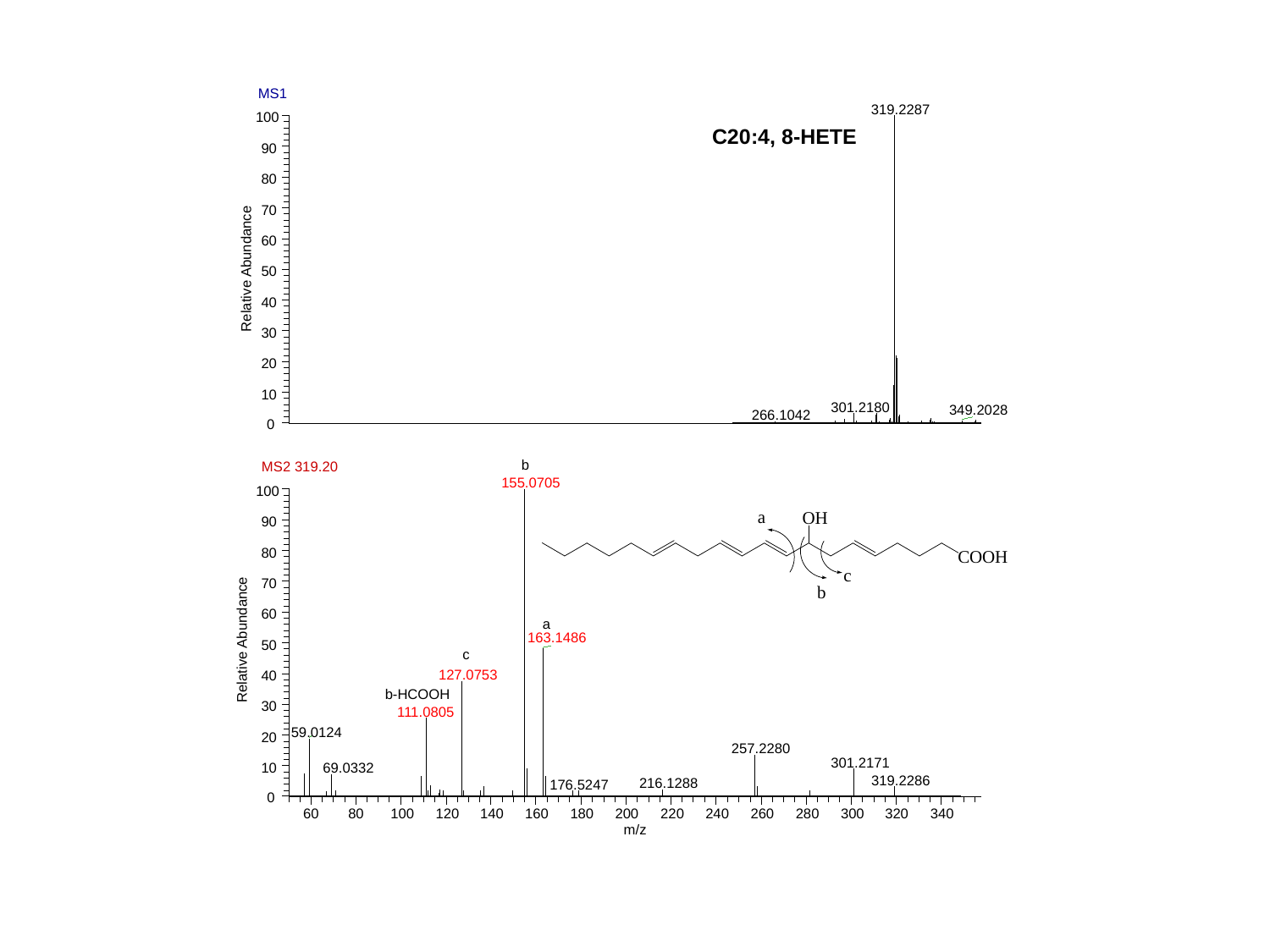

MS1
319.2287
100
90
80
70
60
Relative Abundance
50
40
30
20
10
301.2180
349.2028
266.1042
0
C20:4, 8-HETE
b
MS2 319.20
155.0705
100
90
80
70
60
a
163.1486
50
c
127.0753
40
b-HCOOH
30
111.0805
59.0124
20
257.2280
301.2171
10
69.0332
319.2286
216.1288
176.5247
0
60
80
100
120
140
160
180
200
220
240
260
280
300
320
340
m/z
Relative Abundance

## Slide 12
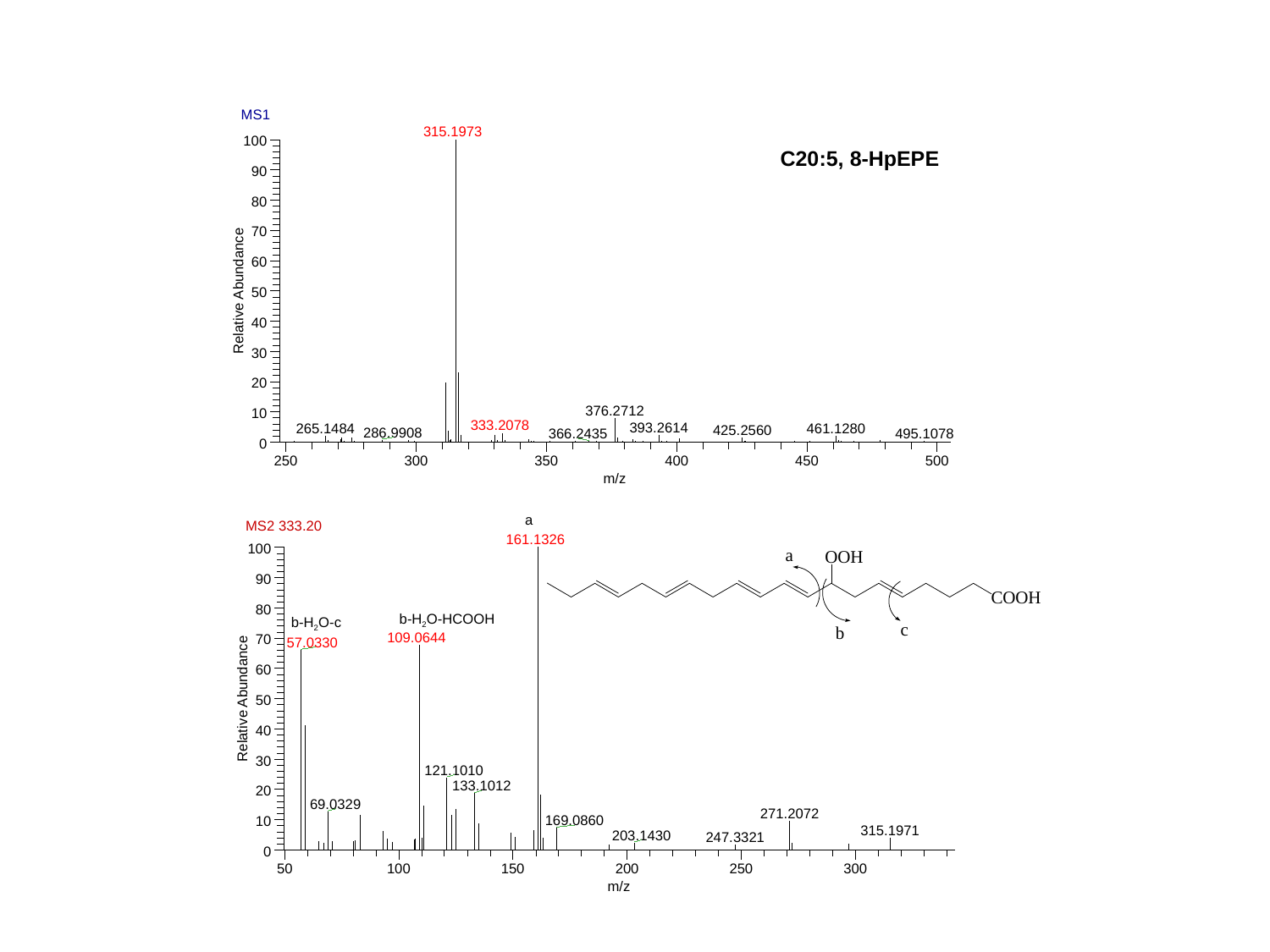

MS1
315.1973
100
90
80
70
60
Relative Abundance
50
40
30
20
376.2712
10
333.2078
393.2614
461.1280
265.1484
425.2560
286.9908
366.2435
495.1078
0
250
300
350
400
450
500
m/z
C20:5, 8-HpEPE
a
MS2 333.20
161.1326
100
90
80
b-H2O-HCOOH
b-H2O-c
109.0644
70
57.0330
60
Relative Abundance
50
40
30
121.1010
133.1012
20
69.0329
271.2072
169.0860
10
315.1971
203.1430
247.3321
0
50
100
150
200
250
300
m/z

## Slide 13
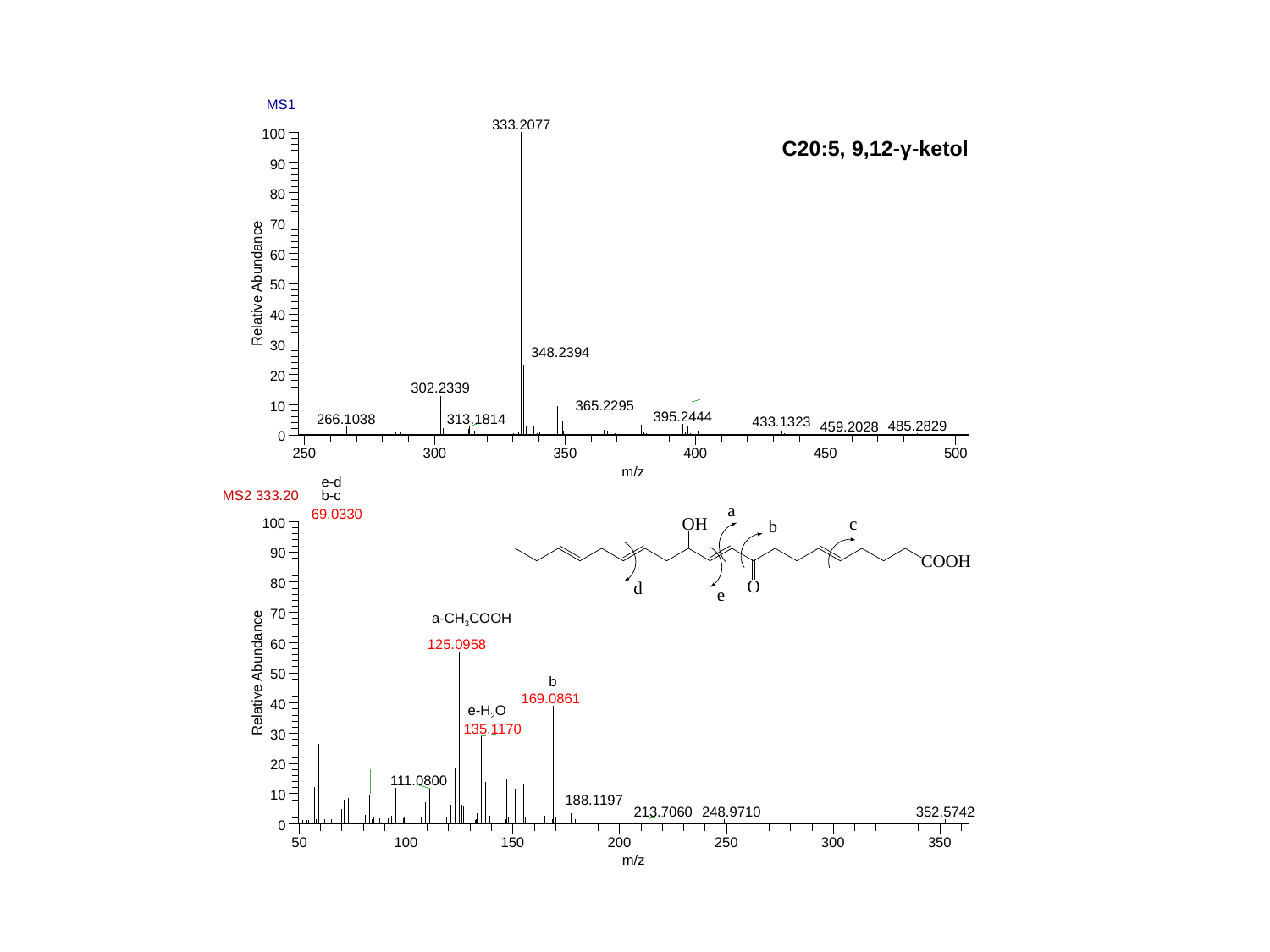

MS1
333.2077
100
90
80
70
60
Relative Abundance
50
40
30
348.2394
20
302.2339
365.2295
10
395.2444
266.1038
313.1814
433.1323
485.2829
459.2028
0
250
300
350
400
450
500
m/z
C20:5, 9,12-γ-ketol
e-d
b-c
MS2 333.20
69.0330
100
90
80
70
a-CH3COOH
60
125.0958
Relative Abundance
50
b
169.0861
40
e-H2O
135.1170
30
20
111.0800
10
188.1197
352.5742
213.7060
248.9710
0
50
100
150
200
250
300
350
m/z

## Slide 14
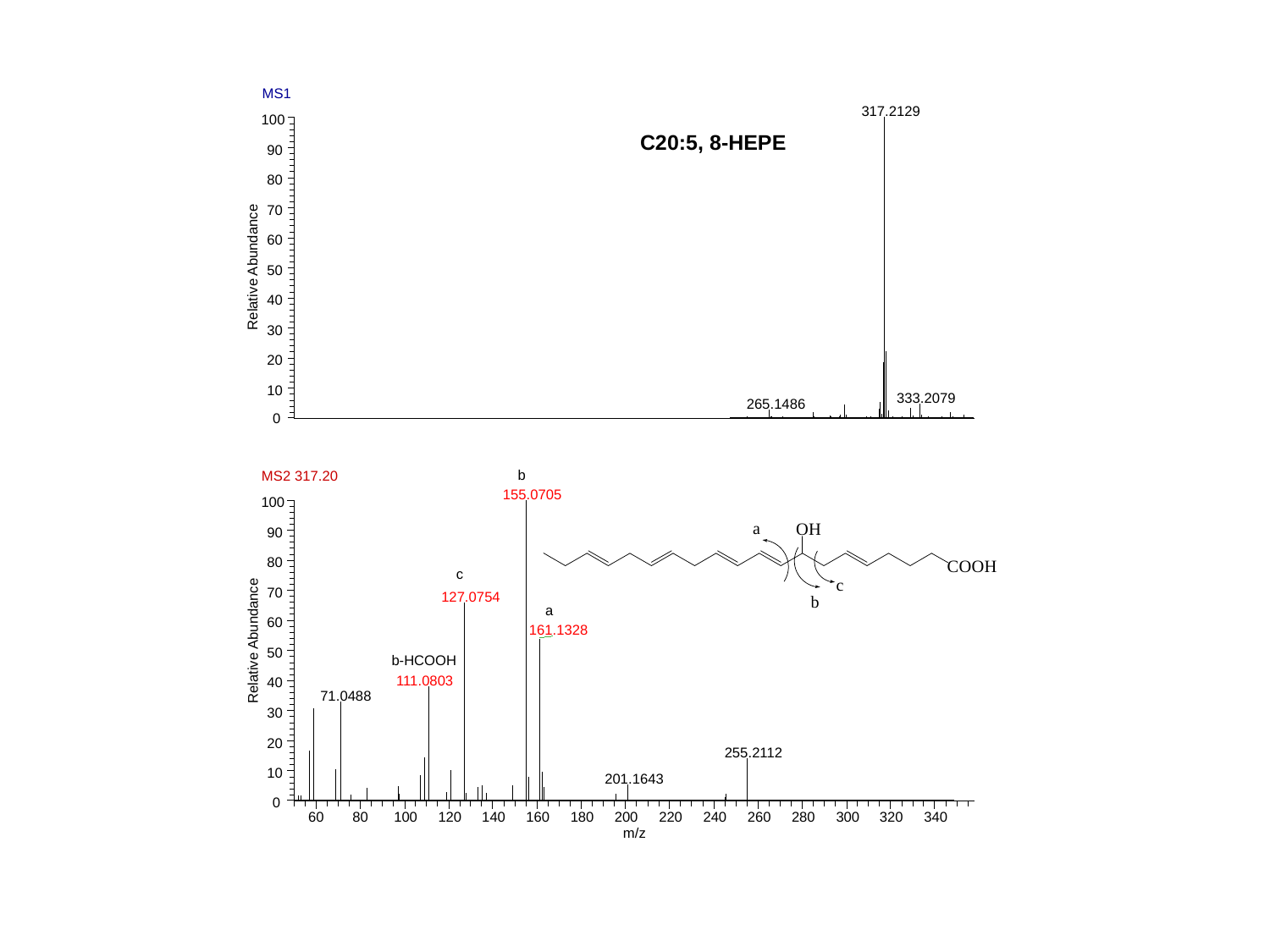

MS1
317.2129
100
90
80
70
60
Relative Abundance
50
40
30
20
10
333.2079
265.1486
0
C20:5, 8-HEPE
b
MS2 317.20
155.0705
100
90
80
c
70
127.0754
a
60
161.1328
50
b-HCOOH
111.0803
40
71.0488
30
20
255.2112
10
201.1643
0
60
80
100
120
140
160
180
200
220
240
260
280
300
320
340
m/z
Relative Abundance
